# A One-Pot Approach to Novel Pyridazine *C*-Nucleosides

**DOI:** 10.3390/molecules26082341

**Published:** 2021-04-17

**Authors:** Flavio Cermola, Serena Vella, Marina DellaGreca, Angela Tuzi, Maria Rosaria Iesce

**Affiliations:** 1 Dipartimento di Scienze Chimiche, Università di Napoli Federico II, Complesso Universitario di M. Sant’Angelo, Via Cintia, 80126 Napoli, Italy; dellagre@unina.it (M.D.); angela.tuzi@unina.it (A.T.); iesce@unina.it (M.R.I.); 2 Erbagil s.r.l., Via L. Settembrini, 13, 82037 Telese Terme, Italy; s.vella@erbagil.com

**Keywords:** glycosyl furans, singlet oxygen, [4+2] cycloaddition, *C*-nucleosides, photooxygenation, pyridazines, reduction

## Abstract

The synthesis of glycosides and modified nucleosides represents a wide research field in organic chemistry. The classical methodology is based on coupling reactions between a glycosyl donor and an acceptor. An alternative strategy for new *C*-nucleosides is used in this approach, which consists of modifying a pre-existent furyl aglycone. This approach is applied to obtain novel pyridazine *C*-nucleosides starting with 2- and 3-(ribofuranosyl)furans. It is based on singlet oxygen [4+2] cycloaddition followed by reduction and hydrazine cyclization under neutral conditions. The mild three-step one-pot procedure leads stereoselectively to novel pyridazine *C*-nucleosides of pharmacological interest. The use of acetyls as protecting groups provides an elegant direct route to a deprotected new pyridazine *C*-nucleoside.

## 1. Introduction

The synthesis of nucleoside analogues has a prominent role in the field of organic chemistry and biology [1]. Among modified nucleosides, *C*-nucleosides represent a special moiety of this compound class due to their higher stability towards enzymatic and chemical hydrolysis than that of natural *N*-nucleosides, as well as owing to the interesting biological and pharmacological properties of some of their derivatives [1,2]. The first natural *C*-nucleoside isolated was pseudouridine [3], followed by showdomycin [4], and oxazinomycin [5]—all characterized by important pharmacological activities, from antitumor to antibacterial. *C*-nucleosides comprise a sugar moiety and a non-natural heterocyclic base connected by a carbon–carbon bond. The stability of this bond and the broad structural variation in the heterocyclic base account for the high interest in this compound class [6,7,8,9]. The main synthetic approach for *C*-glycosides is based on coupling reactions between a glycosyl donor and an acceptor in the presence of a promoter [10]. Alternatively, structural elaborations on a pre-existing aglycone are performed to afford the final *C*-glycoside [10]. In this context, the use of the furan as starting aglycone is of particular interest due to the easy preparation and high versatility of this heterocycle [11]. We elaborated a green procedure based on the use of a furan aglycone as a building block and the [4+2] cycloaddition of singlet oxygen [12] by dye-sensitized photooxygenation as a strategy for providing the final desired structures. Indeed, singlet oxygen addition to furan systems offers various elegant routes to differently functionalized derivatives through structural elaborations of the initial endoperoxides, the 2,3,7-trioxabicyclo[2.2.1]hept-5-enes [13]. Although the furan endoperoxides are thermally unstable, their reactivity can be controlled and opportunely addressed by working at low temperatures. With this strategy, it is possible to synthesize novel glycosyl derivatives, such as glycals [14] and spiroketals of monosaccharides, which are structural motifs of many products characterized by important and assorted biological properties [15]. Pyridazine [16], pyrazoline [14], bis-epoxide [17], and spirocyclic *C*-nucleosides [17] were also obtained. Key intermediates are often unsaturated *Z*-1,4-enediones, easily obtained by photooxygenation and followed by in situ reduction of the corresponding endoperoxides [14,15,16,17] at low temperatures. In many cases, the photochemical approach represents an alternative to other methods. As an example, Maeba described the synthesis of 3-(2′,3′,5′-tri-*O*-benzoyl-β-ribofuranosyl)pyridazine through a series of reactions involving the oxidation of the furan ring by bromine and methanol [18]. We obtained an analogue 4-(2′,3′,5′-tri-*O*-acetyl-β-ribofuranosyl)-3,6-dimetylpyridazine in 70% yield (based on starting furan) by oxygenation, followed by diethyl sulfide reduction and hydrazine hydrochloride cyclization [16]. The efficient formation of this compound prompted us to explore the possibility of extending this approach to novel furans in order to synthesize 3- and 4-(ribofuranosyl)pyridazines. The pyridazine ring is recognized as a versatile pharmacophore. In the last years, particular attention has been devoted to developing novel synthetic approaches to this system, starting with new precursors or by utilizing green methodologies [19,20,21,22].

## 2. Results and Discussion

**Scheme 1:**
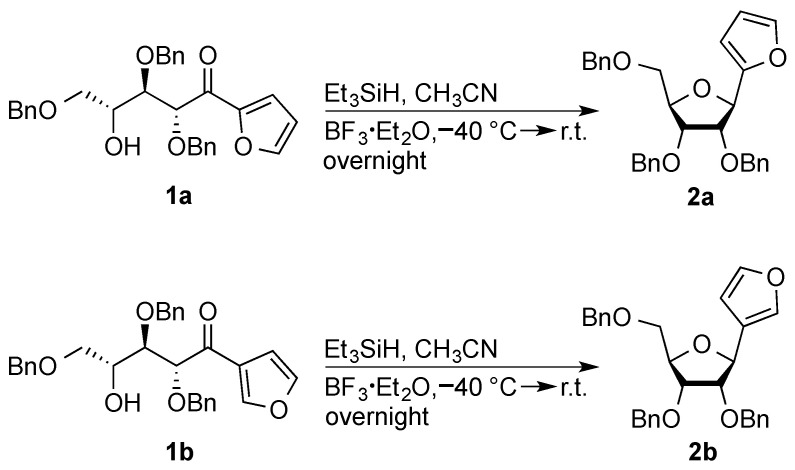
Stereoselective synthesis of 2- and 3-(β-ribofuranosyl)furans **2a**,**b**.

For this purpose, the suitable furans **2a**,**b** were initially prepared by the β-stereoselective reduction [23] of the corresponding furyl ketoses **1a**,**b** (Scheme 1). The latter were obtained by coupling reactions between 2,3,5-tri-*O*-benzyl-d-ribono-1,4-lactone as the glycosyl donor, and 2- or 3-furyllithium, according to a previously reported procedure [15].

**Scheme 2:**
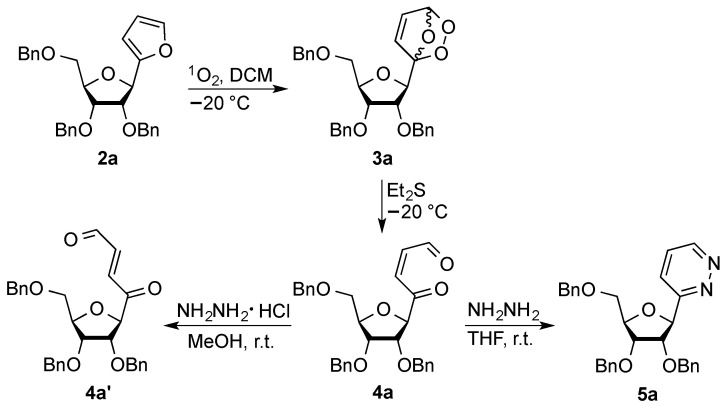
Dye-sensitized photooxygenation of ribofuranosyl furan **2a**, followed by reduction and hydrazine treatment.

The reduction times were particularly long (overnight), probably owing to the open-chain structures of the furyl derivatives **1a**,**b** in equilibrium with very small amounts of the corresponding cyclic structures, as evidenced by their proton spectra. Although the ^1^H-NMR spectra of both crude reaction mixtures showed only the presence of products **2a**,**b**, a considerable loss of material occurred during purification by silica gel chromatography, even if performed under N_2_, as reported in the literature for **2a** [24]. Firstly, **2a** was photo-oxygenated at −20 °C in dichloromethane with methylene blue as the sensitizer (Scheme 2). When the reaction was complete (90 min, TLC or ^1^H-NMR), Et_2_S (1.2 equiv.) was added to the crude mixture, which was kept at −20 °C to avoid thermal rearrangement of the intermediate endoperoxide [12,13,14,15,16,17].

**Scheme 3:**
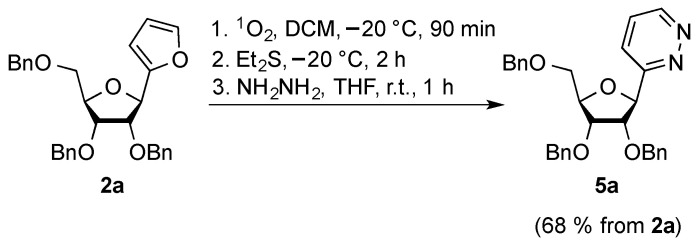
One-pot synthesis of the 3-(2′,3′,5′-tri-*O*-benzyl-β-d-ribofuranosyl)pyridazine (**5a**).

**Figure 1:**
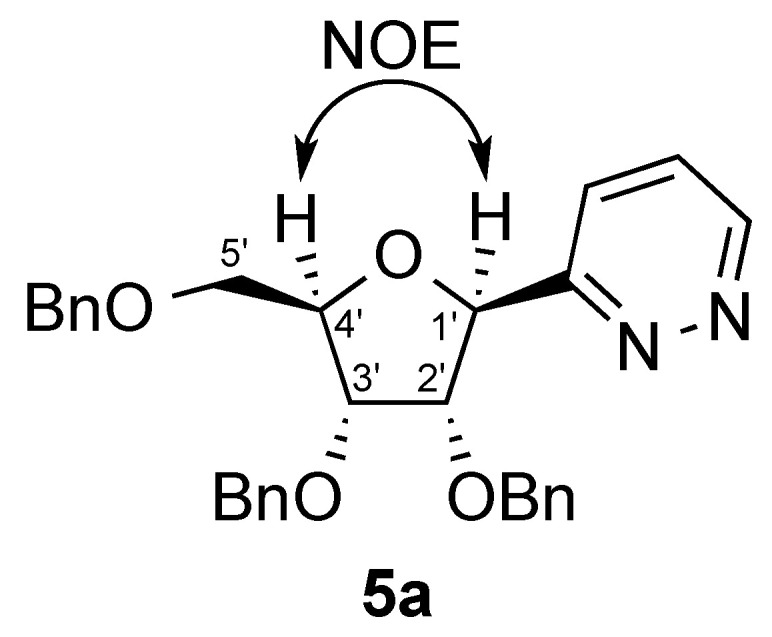
NOESY correlation in **5a**.

After 2 h, the solvent and unreacted Et_2_S were removed under reduced pressure, and the crude **4a** was treated with hydrazine hydrochloride, as reported in [16]. Under these conditions, compound **4a** rapidly isomerized into the more stable *E*-isomer **4a’**, owing to the configurational instability of the Z-enedione **4a** under acidic conditions, as observed in similar cases [12,16] (Scheme 2). However, we found that when a solution of hydrazine in THF (2.0 M) was added to the crude glycosyl enedione **4a**, the expected pyridazine **5a** was promptly obtained (Scheme 2). The synthesis of **5a** was then realized through a one-pot three-step procedure in good total yield, as shown in Scheme 3. The β-configuration of pyridazine **5a** was confirmed by the 2D-NOESY experiment, which evidenced correlation between the H-1′ and the H-4′ of the sugar ring (Figure 1). With the exception of the protecting groups, the 3-(ribofuranosyl)pyridazine **5a** was the same as that reported by Maeba et al. [18].

**Scheme 4:**
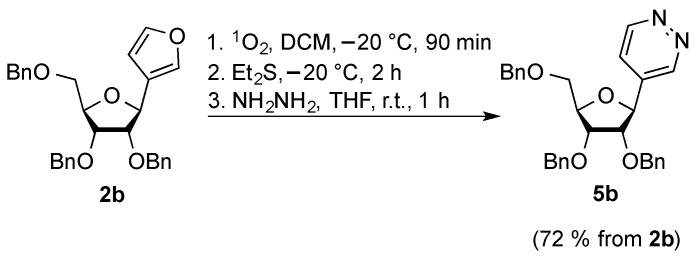
One-pot synthesis of the 4-(2′,3′,5′-tri-*O*-benzyl-β-d-ribofuranosyl)pyridazine (**5b**).

The novel conditions were applied to furan **2b** and, in good total yield, led to the new 4-(ribofuranosyl)pyridazine **5b** (Scheme 4). The β-configuration was also confirmed for **5b** by the 2D-NOESY experiment.

The relevance of the protecting groups in the glycoside chemistry, as well as the possibility of using easily removable groups, motivated us to extend the one-pot route to a sugarfuran protected with acetyls. Hence, we carried out a coupling reaction with the 2,3,5-tetra-1-*O*-acetyl-β-d-ribofuranose as the glycosyl donor and methyl furan-2-carboxylate as the acceptor—both commercially available—in the presence of SnCl_4_ as the promoter, according to a reported procedure [17].

This synthetic approach was described to lead mainly to β-anomers due to the participation of the neighboring acetyl group at the C-2′ of the sugar ring during the departure of the leaving group at C-1′ [25,26].

**Scheme 5:**
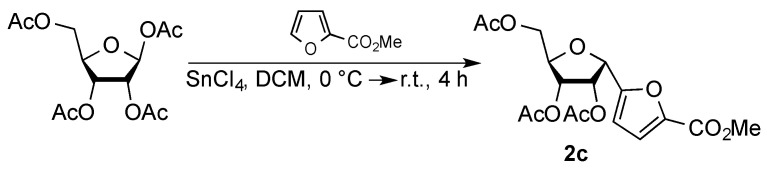
Synthesis of the ribofuranosyl furan α-**2c**.

**Figure 2:**
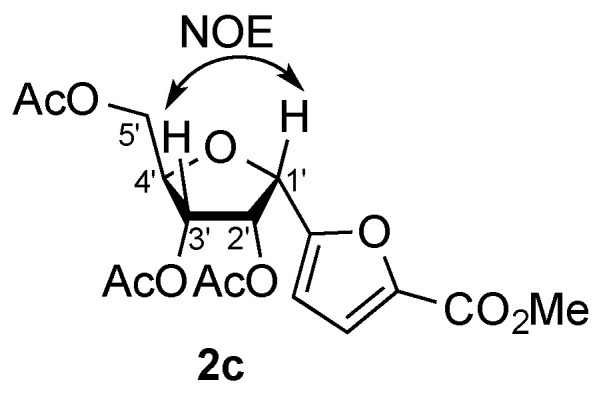
NOESY correlation in **2c**.

Surprisingly, the reaction afforded only the ribofuranosyl furan **2c**, whose α-configuration was assigned by the 2D-NOESY experiment (Scheme 5). Indeed, a NOE effect between the H-1′ and the H-3′ of the sugar ring in C_6_D_6_ was evidenced (Figure 2). In this anisotropic solvent, the H-3′ and H-4′ signals do not overlap as it occurs when the ^1^H-NMR spectrum is recorded in CDCl_3_ (aromatic-solvent-induced shift (ASIS) effects) [27]. The previously incorrect β-configuration for **2c** was assigned on the basis of the constant value of the coupling, in comparison with those of the two anomers of showdomycin, and the synthetic procedure used [17].

Attempts to synthesize the β-anomer of **2c** by carrying out the coupling reaction in acetonitrile and/or by using different promoters (BF_3_ or TMSOTf) failed, and only the α-**1c** was obtained, as evidenced spectroscopically and chromatographically.

A possible rationalization of this unexpected result is that the coupling proceeds here through anchimeric assistance by the acetyl group at the C-5′. A similar stereochemical trend is described in the iodination of some acetylated oxathiolanes [28]. Otherwise, the use of the only β-anomer of 1,2,3,5-tetra-*O*-acetyl-d-ribofuranose as a glycosyl donor together with the observed full stereoselectivity suggest that the reaction to methyl furan-2-carboxylate could occur through a concerted pathway leading to the only α-anomer of **2c**. This hypothesis could be confirmed by the use of the only α-anomer of 1,2,3,5-tetra-*O*-acetyl-d-ribofuranose as a glycosyl donor, which should lead to the β-anomer of **1c**. Control experiments showed that the peracetylation reaction in pyridine or catalyzed by the TMSOTf afforded the peracetylated pyranosic form of the sugar as the main product, as well as the β-anomer of 1,2,3,5-tetra-*O*-acetyl-d-ribofuranose, which is the only commercially available form.

**Scheme 6:**
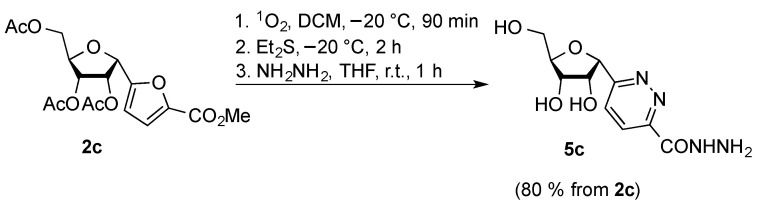
One-pot synthesis of the 6-(α-d-ribofuranosyl)pyridazine-3-carbohydrazide (**5c**).

The mild procedure for pyridazine C-nucleoside was later applied to the 2-(2′,3′,5′-tri-*O*-acetyl-α-d-ribofuranosyl)furan (**2c**). Unexpectedly, the sequence of reactions on **2c** led in high yield to the deprotected pyridazine C-nucleoside **5c** (Scheme 6).

**Scheme 7:**
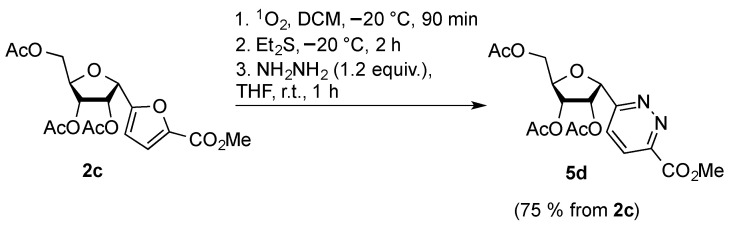
One-pot synthesis of the 3-(2′,3′,5′-tri-*O*-acetyl-α-d-ribofuranosyl)-6-(methoxycarbonyl)pyridazine (**5d**).

With the aim of preserving the methyl ester function, as well as the protecting acetyl groups on the sugar ring, the reaction was carried out under the same conditions except for the use of 1.2 equiv. of hydrazine. In this case, the one-pot procedure afforded the protected pyridazine C-nucleoside **5d** good yield, showing that the cyclization proceeds more rapidly than the attack to the ester functions (Scheme 7).

**Figure 3:**
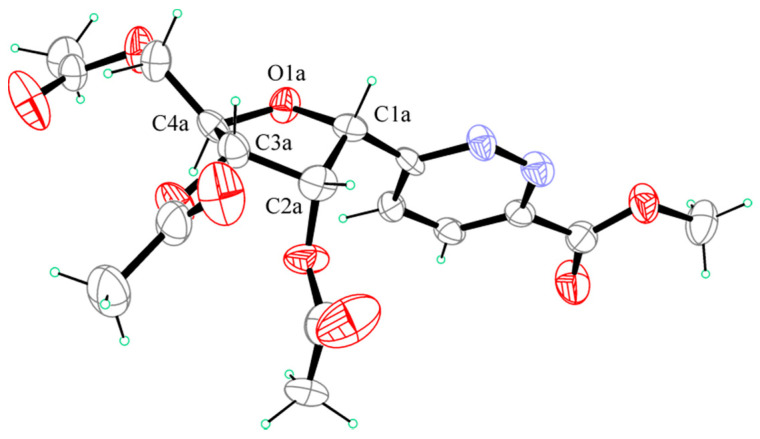
Molecular structure of **5d** showing the α-configuration (Ortep view with ellipsoids drawn at 30% probability level).

The structure of compound **5d**, assigned by NMR data, was confirmed by X-ray crystallographic analysis (Figure 3).

**Scheme 8:**
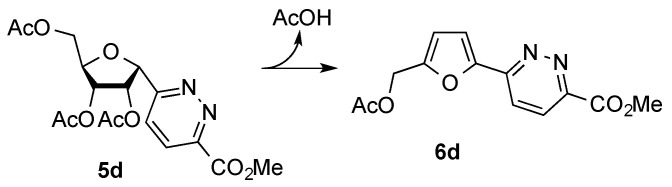
Aromatization of **5d** into **6d**.

Pyridazine **5d** was quite stable; however, it slowly aromatized after several days at room temperature, leading to the corresponding furan **6d** (Scheme 8). Similar elimination was previously observed in a benzoylated pyridazine C-nucleoside [18].

## 3. Materials and Methods

### 3.1. General Information

Melting points are uncorrected. The ^1^H- and ^13^C-NMR spectra were recorded at 500 and 125 MHz, respectively, on a Fourier Transform NMR Varian 500 Unity Inova spectrometer. The carbon multiplicity was evidenced by DEPT experiments. The proton couplings were evidenced by ^1^H-^1^H COSY experiments. The heteronuclear chemical shift correlations were determined by HMQC and HMBC pulse sequences. ^1^H-^1^H proximities through space within a molecule were determined by NOESY experiments. X-ray analysis was performed on a Bruker-Nonius Kappa CCD (Nonius BV, Delft, The Netherlands) diffractometer (graphite monochromated Mo K_α_ radiation, λ = 0.71073 Å, CCD rotation images, thick slices, φ and ω scans to fill asymmetric unit). Analytical TLC was performed on precoated silica gel plates (Macherey-Nagel, Düren, Germany) with 0.2 mm film thickness. Spots were visualized by UV light and by spraying with EtOH/H_2_SO_4_ (95:5 *v*/*v*), followed by heating for 5 min at 110 °C. Column chromatography was performed on silica gel (0.063–0.2 mm) (Macherey-Nagel). Reagent-grade commercially available reagents and solvents were used.

### 3.2. Synthesis of ***2a**,**b***

A solution of 243 mg (0.5 mmol) of **1a** [15] in 5.2 mL of acetonitrile, cooled to −40 °C, was added to 240 µL (3 equiv.) of triethylsilane and 70 µL (1 equiv.) of BF_3_·Et_2_O. The solution was stirred at −40 °C for 4 h, which was allowed to rise to r.t. while the mixture was further stirred overnight. A saturated aqueous solution of K_2_CO_3_ was later added (10 mL), and the mixture was kept under stirring for 10 min. The organic layer was extracted with diethyl ether (3 × 30 mL), washed with brine, dried over anhydrous Na_2_SO_4_, and filtered. The solvent was removed under reduced pressure, and the residue was chromatographed on flash silica gel (*n*-hexane/ether 1:1 *v*/*v*), affording the *C*-nucleoside β-**2a** [24] with 35% yield.

β-**2a**: oil; ^1^H-NMR; δ = 3.61 (m, 2 H, H-5′_A_ and H-5′_B_), 4.05 (dd, *J* = 4.9 Hz, 1 H, H-3′), 4.18 (dd, *J* = 6.5, 4.9 Hz, 1 H, H-2′), 4.30 (m, 1 H, H-4′), 4.50–4.66 (m, 6 H, CH_2_ of Bn), 5.04 (d, *J* = 6.5 Hz, 1 H, H-1′), 6.34 (bs, 2 H, H-3 and H-4), 7.23–7.33 (m, 15 H, 3× Ph), 7.34 (bs, 1 H, H-5); ^13^C-NMR; δ = 70.3 (t), 72.1 (2× t), 73.4 (t), 76.5 (d), 77.7 (d), 79.9 (d), 81.5 (d), 108.9 (d), 110.3 (d), 127.5 (d), 127.6 (d), 127.7 (d), 127.8 (d), 128.0 (d), 128.3 (d), 137.7 (s), 138.0 (s), 138.2 (s), 142.5 (d), 152.2 (s).

The synthesis of **2b** was performed as reported above for **2a**, starting from **1b** [15]. The flash silica gel (*n*-hexane/ether 1:1 *v*/*v*) afforded the novel *C*-nucleoside β-**2b** with 30% yield.

β-**2b**: mp 49–51 °C; ^1^H-NMR; δ = 3.56 (dd, *J* = 10.4, 4.4 Hz, 1 H, H-5′_A_), 3.59 (dd, *J* = 10.4, 4.4 Hz, 1 H, H-5′_B_), 3.84 (dd, *J* = 6.6, 4.9 Hz, 1 H, H-2′), 3.99 (dd, *J* = 4.9, 3.8 Hz, 1 H, H-3′), 4.28 (m, 1 H, H-4′), 4.48–4.62 (m, 6 H, CH_2_ of Bn), 4.97 (d, *J* = 6.6 Hz, 1 H, H-1′), 6.31 (bs, 1 H, H-4), 7.22–7.33 (m, 15 H, 3× Ph), 7.35 (bs, 1 H, H-5), 7.40 (s, 1 H, H-2); ^13^C-NMR; δ = 70.4 (t), 71.9 (t), 72.2 (t), 73.4 (t), 75.7 (d), 77.5 (d), 81.6 (d), 82.2 (d), 108.5 (d), 124.4 (s), 127.6 (d), 127.7 (d), 127.8 (d), 128.0 (d), 128.3 (d), 137.7 (s), 137.9 (s), 138.1 (s), 140.2 (d), 143.2 (d). HRMS (ESI-TOF) calcd. for C_30_H_31_O_5_ [M + H]^+^ 471.2171, found 471.2168. Anal. Calcd. for C_30_H_30_O_5_: C, 76.57; H, 6.43. Found: C, 76.45; H, 6.52.

### 3.3. Synthesis of Pyridazines: General Procedure

A 0.02 M solution of **2** (0.25 mmol) in dry CH_2_Cl_2_ was irradiated at −20 °C with a halogen lamp (650 W) in the presence of methylene blue (MB, 1 × 10^−3^ mmol), while dry oxygen was bubbled through the solution. The progress of each reaction was checked by periodically monitoring (TLC, or ^1^H-NMR) the disappearance of **2**. When the reaction was complete (ca. 90 min), 1.2 equiv. of Et_2_S were added to the crude solution, which was kept at −20 °C for 2 h. Thus, the solvent and the unreacted Et_2_S were removed under reduced pressure, and 1 mL of a hydrazine solution in THF (2 M) was added (1.2 equiv. for **5d**). The resulting mixture was kept under stirring at r.t. for 1 h. Subsequently, the solvent and the excess hydrazine were removed under reduced pressure to afford the crude product **5**. Silica gel chromatography (*n*-hexane/ether) afforded the pure pyridazine derivatives **5**. 

*3-(2′,3′,5′-tri-O-Benzyl-β-d-ribofuranosyl)pyridazine* (**5a**), (68% yield, from **2a**); oil; ^1^H-NMR (CDCl_3_); δ = 3.66 (dd, *J* = 10.4, 3.3 Hz, 1 H, H-5′_A_), 3.88 (dd, *J* = 10.4, 2.5 Hz, 1 H, H-5′_B_), 3.97 (dd, *J* = 7.7, 4.9 Hz, 1 H, H-3′), 4.31 (dd, *J* =4.9, 2.7 Hz, 1 H, H-2′), 4.36 (d, *J* = 12.0 Hz, 1 H, CH of Bn), 4.44 (m, 1 H, H-4′), 4.50 (d, *J* = 11.5 Hz, 1 H, CH of Bn), 4.56 (d, *J* = 12.0 Hz, 1 H, CH of Bn), 4.57 (d, *J* = 11.5 Hz, 1 H, CH of Bn), 4.74 (d, *J* = 12.0 Hz, 1 H, CH of Bn), 4.86 (d, *J* = 12.0 Hz, 1 H, CH of Bn), 5.48 (d, *J* = 2.7 Hz, 1 H, H-1′) 7.20 (dd, *J* = 8.8, 4.9 Hz, 1 H, H-5), 7.22–7.40 (m, 15 H, 3× Ph), 7.80 (dd, *J* = 8.8, 1.6 Hz, 1 H, H-4), 9.03 (dd, *J* = 4.9, 1.6 Hz, 1 H, H-6); ^13^C-NMR; δ = 69.1 (t), 71.7 (t), 72.1 (t), 73.4 (t), 76.3 (d), 80.9 (d), 81.5 (d), 83.3 (d), 124.9 (d), 126.6 (d), 127.7 (d), 127.8 (d), 128.2 (d), 128.3 (d), 137.7 (s), 138.0 (s), 150.4 (d), 162.8 (s). HRMS (ESI-TOF) calcd. for C_30_H_31_N_2_O_4_ [M + H]^+^ 483.2284, found 483.2280. Anal. Calcd. for C_30_H_30_N_2_O_4_: C, 74.67; H, 6.27; N 5.81. Found: C, 74.56; H, 6.35; N 5.72.

*4-(2′,3′,5′-tri-O-Benzyl-β-d-ribofuranosyl)pyridazine* (**5b**), (72% yield, from **2b**); oil; ^1^H-NMR (CDCl_3_); δ = 3.57 (dd, *J* = 10.4, 3.3 Hz, 1 H, H-5′_A_), 3.65 (dd, *J* = 10.4, 3.8 Hz, 1 H, H-5′_B_), 3.77 (dd, *J* = 7.7, 4.9 Hz, 1 H, H-2′), 4.02 (dd, *J* = 4.9, 2.7 Hz, 1 H, H-3′), 4.38 (m, 2H, H-4′ and CH of Bn), 4.51 (d, *J* = 12.0 Hz, 1 H, CH of Bn), 4.56 (d, *J* = 12.0 Hz, 1 H, CH of Bn), 4.57(d, *J* = 12.0 Hz, 1 H, CH of Bn), 4.60 (s, 2 H, CH_2_ of Bn), 4.98 (d, *J* = 7.7 Hz, 1 H, H-1′), 7.17–7.35 (m, 15 H, 3 x Ph), 7.47 (bd, *J* = 5.5 Hz 1 H, H-5), 9.00 (d, *J* = 5.5 Hz, 1 H, H-6), 9.16 (bs, 1 H, H-3); ^13^C-NMR; δ = 70.1 (t), 72.0 (t), 72.7 (t), 73.6 (t), 77.1 (d), 78.6 (d), 82.7 (d), 83.4 (d), 123.3 (d), 127.7 (d), 127.9 (d), 128.0 (d), 128.1 (d), 128.5 (d), 136.9 (s), 137.4 (s), 137.6 (s), 140.3 (s), 149.7 (d), 151.0 (d). HRMS (ESI-TOF) calcd. for C_30_H_31_N_2_O_4_ [M + H]^+^ 483.2284, found 483.2281. Anal. Calcd. for C_30_H_30_N_2_O_4_: C, 74.67; H, 6.27; N 5.81. Found: C, 74.53; H, 6.37; N 5.69.

*6-(α-d-Ribofuranosyl)pyridazine-3-carbohydrazide* (**5c**), (80% yield, from **2c**); m.p. 215–217 °C; ^1^H-NMR (DMSO); δ = 3.49 (m, 1 H, H-5′_A_), 3.66 (m, 1 H, H-5′_B_), 3.98 (m, 1 H, H-4′), 4.19 (m, 2 H, H-3′ and H-2′), 4.77 (t, *J* = 4.8 Hz, 1 H, OH), 4.93 (d, *J* = 4.4 Hz, 1 H, OH), 5.00 (d, *J* = 6.6 Hz, 1 H, OH), 5.33 (d, *J* = 3.4 Hz, 1 H, H-1′), 7.80 (d, *J* = 8.6 Hz, 1 H, H-5), 8.11 (d, *J* = 8.6 Hz, 1 H, H-4); ^13^C-NMR (DMSO); δ = 61.9 (t), 72.9 (d), 73.9 (d), 82.3 (d), 83.5 (d), 125.3 (d), 128.1 (d), 152.6 (s), 162.0 (s), 164.2 (s). HRMS (ESI-TOF) calcd. for C_10_H_15_N_4_O_5_ [M + H]^+^ 271.1042, found 271.1040. Anal. Calcd. for C_10_H_14_N_4_O_5_: C, 44.44; H, 5.22; N 20.73. Found: C, 44.33; H, 5.30; N 20.69.

*3-(2′,3′,5′-tri-O-Acetyl-α-d-ribofuranosyl)-6-(methoxycarbonyl)pyridazine* (**5d**), (75% yield, from **1c**); m.p. 124–126 °C; ^1^H-NMR (CDCl_3_); δ = 1.81 (s, 3 H, CH_3_CO), 2.02 (s, 3 H, CH_3_CO), 2.14 (s, 3 H, CH_3_CO), 4.09 (s, 3 H, OCH_3_), 4.23 (dd, *J* = 11.7, 3.9 Hz, 1 H, H-5′_A_), 4.49 (m, 2 H, H-5′_B_ and H-4′), 5.56 (dd, *J* = 7.4, 4.0 Hz, 1 H, H-3′), 5.78 (d, *J* = 4.0 Hz, 1 H, H-1′), 5.94 (dd, *J* = 4.0 Hz, 1 H, H-2′), 7.89 (d, *J* = 7.4 Hz, 1 H, H-5), 8.22 (d, *J* = 7.4 Hz, 1 H, H-4); ^13^C-NMR (CDCl_3_); δ = 17.0 (q), 20.4 (q), 20.8 (q), 53.4 (q), 63.3 (t), 72.2 (d), 73.4 (d), 78.4 (d), 80.4 (d), 125.8 (d), 127.5 (d), 150.9 (s), 162.0 (s), 164.6 (s), 168.8 (s), 169.5 (s), 170.6 (s). HRMS (ESI-TOF) calcd. for C_17_H_21_N_2_O_9_ [M + H]^+^ 397.1247, found 397.1244. Anal. Calcd. for C_17_H_20_N_2_O_9_: C, 51.52; H, 5.09; N, 7.07. Found: C, 52.33; H, 5.20; N 7.18.

### 3.4. Aromatization of ***5d***

Crystals of **5d** (25 mg) were kept without solvent at r.t. for one week. A ^1^H-NMR spectrum was then recorded, which showed the presence of the furan **6d** and traces of acetic acid. TLC chromatography (*n*-hexane/ether 6:4 *v*/*v*) afforded the furan **6d** as solid in 85% yield.

m.p. 169–170 °C; ^1^H-NMR (CDCl_3_); δ = 2.12 (s, 3 H, CH_3_CO), 4.08 (s, 3 H, OCH_3_), 5.16 (s, 2 H, CH_2_), 6.64 (d, *J* = 3.5 Hz, 1 H, H-4′), 7.49 (d, *J* = 3.5 Hz, 1 H, H-3′), 7.95 (d, *J* = 8.7 Hz, 1 H, H-5), 8.19 (d, *J* = 8.7 Hz, 1 H, H-4); ^13^C-NMR (CDCl_3_); δ = 20.8 (q), 53.2 (q), 57.9 (t), 113.6 (d), 113.7 (d), 121.6 (d), 127.9 (d), 149.4 (s), 150.7 (s), 152.7 (s), 153.1 (s), 164.5 (s), 170.4 (s). HRMS (ESI-TOF) calcd. for C_13_H_13_N_2_O_5_ [M + H]^+^ 277.0824, found 277.0822. Anal. Calcd. for C_13_H_12_N_2_O_5_: C, 56.52; H, 4.38; N, 10.14. Found: C, 56.38; H, 4.45; N 10.09.

### 3.5. X-ray Crystallography of ***5d***

X-ray analysis was performed on single crystals of **5d** obtained as colorless blocks by slow evaporation of a DCM/hexane solution at room temperature. One selected crystal was mounted at ambient temperature on a Bruker-Nonius KappaCCD diffractometer (graphite monochromated Mo K_α_ radiation, λ = 0.71073 Å, CCD rotation images, thick slices, *φ* and *ω* scans to fill asymmetric unit). Semiempirical absorption corrections (SADABS [29]) were applied. The structure was solved by direct methods (SIR97 program [30]) and anisotropically refined by the full matrix least-squares method on *F*^2^ against all independently measured reflections using SHELXL-2018/3 To SHELXL (version 2018/3) [31] and WinGX software (version 2014.1) [32]. All of the hydrogen atoms were introduced in calculated positions and refined according to a riding model with C–H distances in the range of 0.93–0.98 Å and with *U*iso (H) equal to 1.2 Ueq or 1.5 Ueq (C_methyl_) of the carrier atom. Compound **5d** crystallizes in P 2_1_ 2_1_ 2_1_ space group with two independent molecules in the asymmetric unit (see Figure S31 of Supporting Information). The two molecules are very similar to each to the other; in molecule A, one acetyl group is split into two positions with refined occupancy factors of 0.61 and 0.39. Some restraints were introduced in the last stage of refinement to regularize the geometry. In the absence of strong anomalous scatterer atoms, the Flack parameter is meaningless, so it was not possible to assign the absolute configuration by anomalous dispersion effects with diffraction measurements on the crystal. The absolute configuration has been assigned by reference to unchanging chiral centers in the synthetic procedure. A rather high residual electronic density (0.472 eA^−3^) is explained by residual thermal disorder of some acetyl groups at ambient temperature. Unfortunately, it was not possible to re-collect data at low temperatures. Crystal data and structure refinement details are reported in Table S1 of SI. Figures were generated using program Ortep-3 [32]. CCDC-1493362.

## 4. Conclusions

In summary, we highlight that novel pyridazine *C*-nucleosides can be easily obtained starting with protected ribofuranosyl furans. Appropriate mild conditions to preserve the *Z*-configuration of the intermediate 1,4-dicarbonyl compound, necessary for the final step that involves the reaction with hydrazine. It is noteworthy that the use of **2a** and **2b** provides the corresponding 3- and 4-(ribofuranosyl)pyridazines **5a** and **5b**, respectively, which would be hard to synthesize through direct coupling reactions with complete regioselectivity. Moreover, the use of an acetylated sugar provides a direct route to deprotected pyridazine-*C*-nucleosides. The three-step one-pot procedure is completely stereoselective and gives the product **5** the same anomeric configuration as the starting sugar furans. 

The ease of methodology, together with the good yields of pyridazine-*C*-nucleosides **5**, foresee novel applications in the field of *C*-nucleosides synthesis since pyridazinesare useful intermediates for constructing heterocycle derivatives [33]. These systems are considered by GlaxoSmithKline as one of the “most developable” heteroaromatic rings [34] and are proposed as privileged structures for drug design [35,36]. Moreover, a broad array of significant biological activities has been evidenced in several compounds with pyridazine rings [37,38,39].

## Data Availability

Data are contained within the article or the supplementary data file.

## Supplementary Materials

The following are available online. ^1^H- and ^13^C-NMR, COSY and NOESY spectra for all new compounds. X-ray crystallographic data for **5d**. Supplementary data associated with this article can be found in the online version, at CCDC-1493362, which contains the supplementary crystallographic data for this paper. These data can be obtained free of charge from The Cambridge Crystallographic Data Centre via www.ccdc.cam.ac.uk/data_request/cif.

## Sample Availability

Samples of the compounds are not available from the authors.
