# Supplementary material for: A One-Pot Approach to Novel Pyridazine C-Nucleosides
Source: Molecules. 2021 Apr 17;26(8):2341. doi: 10.3390/molecules26082341 (PMC8074166; doi:10.3390/molecules26082341)

checkCIF/PLATON report

Structure factors have been supplied for datablock(s) I

THIS REPORT IS FOR GUIDANCE ONLY. IF USED AS PART OF A REVIEW PROCEDURE FOR PUBLICATION, IT SHOULD NOT REPLACE THE EXPERTISE OF AN EXPERIENCED CRYSTALLOGRAPHIC REFEREE.

No syntax errors found.   CIF dictionary   Interpreting this report

Datablock: I

```
Bond precision:  C-C = 0.0147 A          Wavelength=0.71073

Cell:              a=5.783(2)      b=20.372(6)      c=33.116(8)
                   alpha=90        beta=90          gamma=90
Temperature:       298 K

                Calculated                 Reported
Volume          3901(2)                    3901(2)
Space group     P 21 21 21                 P 21 21 21
Hall group      P 2ac 2ab                  P 2ac 2ab
Moiety formula  C17 H20 N2 O9              C17 H20 N2 O9
Sum formula     C17 H20 N2 O9              C17 H20 N2 O9
Mr              396.35                     396.35
Dx,g cm-3       1.350                      1.350
Z               8                          8
Mu (mm-1)       0.111                      0.111
F000            1664.0                     1664.0
F000'           1665.06
h,k,lmax        6,24,39                    6,23,39
Nref            6866[ 3961]                6519
Tmin,Tmax       0.983,0.997                0.852,0.897
Tmin'           0.946

Correction method= # Reported T Limits: Tmin=0.852 Tmax=0.897
AbsCorr = MULTI-SCAN

Data completeness= 1.65/0.95      Theta(max)= 25.013

R(reflections)= 0.0845( 2828)     wR2(reflections)= 0.2228( 6519)

S = 1.014                 Npar= 541
```

The following ALERTS were generated. Each ALERT has the format
test-name_ALERT_alert-type_alert-level.
Click on the hyperlinks for more details of the test.

Alert level B

```
PLAT340_ALERT_3_B Low Bond Precision on  C-C Bonds ..............    0.01465 Ang.
```

Alert level C

```
STRVA01_ALERT_4_C          Flack parameter is too small
           From the CIF: _refine_ls_abs_structure_Flack   -0.500
           From the CIF: _refine_ls_abs_structure_Flack_su    1.000
PLAT026_ALERT_3_C Ratio Observed / Unique Reflections (too) Low ..        43% Check
PLAT089_ALERT_3_C Poor Data / Parameter Ratio (Zmax < 18) ........       7.21 Note
PLAT213_ALERT_2_C Atom C11A           has ADP max/min Ratio .....        3.1 prolat
PLAT220_ALERT_2_C NonSolvent   Resd 2  O   Ueq(max)/Ueq(min) Range        3.2 Ratio
PLAT234_ALERT_4_C Large Hirshfeld Difference O1A      --C1A      .       0.16 Ang.
PLAT234_ALERT_4_C Large Hirshfeld Difference O4A      --C8A      .       0.21 Ang.
PLAT234_ALERT_4_C Large Hirshfeld Difference O6A      --C10A     .       0.22 Ang.
PLAT234_ALERT_4_C Large Hirshfeld Difference O2B      --C5B      .       0.18 Ang.
PLAT234_ALERT_4_C Large Hirshfeld Difference C6B      --C7B      .       0.21 Ang.
PLAT242_ALERT_2_C Low    'MainMol' Ueq as Compared to Neighbors of        C6A Check
PLAT242_ALERT_2_C Low    'MainMol' Ueq as Compared to Neighbors of       C10B Check
PLAT906_ALERT_3_C Large K Value in the Analysis of Variance ......      9.667 Check
PLAT906_ALERT_3_C Large K Value in the Analysis of Variance ......      2.043 Check
PLAT906_ALERT_3_C Large K Value in the Analysis of Variance ......      3.084 Check
PLAT910_ALERT_3_C Missing # of FCF Reflection(s) Below Theta(Min).         10 Note
PLAT911_ALERT_3_C Missing FCF Refl Between Thmin & STh/L=    0.595         47 Report
```

Alert level G

```
PLAT002_ALERT_2_G Number of Distance or Angle Restraints on AtSite         16 Note
PLAT003_ALERT_2_G Number of Uiso or Uij Restrained non-H Atoms ...          6 Report
PLAT032_ALERT_4_G Std. Uncertainty on Flack Parameter Value High .      1.000 Report
PLAT172_ALERT_4_G The CIF-Embedded .res File Contains DFIX Records          2 Report
PLAT175_ALERT_4_G The CIF-Embedded .res File Contains SAME Records          2 Report
PLAT186_ALERT_4_G The CIF-Embedded .res File Contains ISOR Records          1 Report
PLAT301_ALERT_3_G Main Residue  Disorder .............(Resd  1  )        11% Note
PLAT380_ALERT_4_G Incorrectly? Oriented X(sp2)-Methyl Moiety .....       C11A Check
PLAT432_ALERT_2_G Short Inter X...Y Contact  O3A      ..C8B              2.99 Ang.
                                             5/2-x,-y,1/2+z  =      2_755 Check
PLAT720_ALERT_4_G Number of Unusual/Non-Standard Labels ..........         16 Note
PLAT721_ALERT_1_G Bond    Calc     0.97000, Rep     0.96000 Dev...       0.01 Ang.
              C11X     -H11G            1.555   1.555 ........     #   97 Check
PLAT791_ALERT_4_G Model has Chirality at C1A         (Sohnke SpGr)          R Verify
PLAT791_ALERT_4_G Model has Chirality at C1B         (Sohnke SpGr)          R Verify
PLAT791_ALERT_4_G Model has Chirality at C2A         (Sohnke SpGr)          S Verify
PLAT791_ALERT_4_G Model has Chirality at C2B         (Sohnke SpGr)          S Verify
PLAT791_ALERT_4_G Model has Chirality at C3A         (Sohnke SpGr)          R Verify
PLAT791_ALERT_4_G Model has Chirality at C3B         (Sohnke SpGr)          R Verify
PLAT791_ALERT_4_G Model has Chirality at C4A         (Sohnke SpGr)          R Verify
PLAT791_ALERT_4_G Model has Chirality at C4B         (Sohnke SpGr)          R Verify
PLAT860_ALERT_3_G Number of Least-Squares Restraints .............         50 Note
PLAT883_ALERT_1_G No Info/Value for _atom_sites_solution_primary .     Please Do !
PLAT916_ALERT_2_G Hooft y and Flack x Parameter Values Differ by .       0.50 Check
PLAT941_ALERT_3_G Average HKL Measurement Multiplicity ...........        4.8 Low
PLAT965_ALERT_2_G The SHELXL WEIGHT Optimisation has not Converged     Please Check
PLAT978_ALERT_2_G Number C-C Bonds with Positive Residual Density.          1 Info
```

0 **ALERT level A** = Most likely a serious problem - resolve or explain
1 **ALERT level B** = A potentially serious problem, consider carefully
17 **ALERT level C** = Check. Ensure it is not caused by an omission or oversight
25 **ALERT level G** = General information/check it is not something unexpected

```
 2 ALERT type 1 CIF construction/syntax error, inconsistent or missing data
10 ALERT type 2 Indicator that the structure model may be wrong or deficient
11 ALERT type 3 Indicator that the structure quality may be low
20 ALERT type 4 Improvement, methodology, query or suggestion
 0 ALERT type 5 Informative message, check
```

---

checkCIF publication errors

Alert level A

```
PUBL004_ALERT_1_A  The contact author's name and address are missing,
            _publ_contact_author_name and _publ_contact_author_address.
PUBL005_ALERT_1_A  _publ_contact_author_email, _publ_contact_author_fax and
            _publ_contact_author_phone are all missing.
            At least one of these should be present.
PUBL006_ALERT_1_A  _publ_requested_journal is missing
            e.g. 'Acta Crystallographica Section C'
PUBL008_ALERT_1_A  _publ_section_title is missing. Title of paper.
PUBL009_ALERT_1_A  _publ_author_name is missing. List of author(s) name(s).
PUBL010_ALERT_1_A  _publ_author_address is missing. Author(s) address(es).
PUBL012_ALERT_1_A  _publ_section_abstract is missing.
            Abstract of paper in English.
```

Alert level G

```
PUBL017_ALERT_1_G The _publ_section_references section is missing or
            empty.
```

```
 7 ALERT level A = Data missing that is essential or data in wrong format
 1 ALERT level G = General alerts. Data that may be required is missing
```

Publication of your CIF

You should attempt to resolve as many as possible of the alerts in all categories. Often the minor alerts point to easily fixed oversights, errors and omissions in your CIF or refinement strategy, so attention to these fine details can be worthwhile. In order to resolve some of the more serious problems it may be necessary to carry out additional measurements or structure refinements. However, the nature of your study may justify the reported deviations from journal submission requirements and the more serious of these should be commented upon in the discussion or experimental section of a paper or in the "special_details" fields of the CIF. *checkCIF* was carefully designed to identify outliers and unusual parameters, but every test has its limitations and alerts that are not important in a particular case may appear. Conversely, the absence of alerts does not guarantee there are no aspects of the results needing attention. It is up to the individual to critically assess their own results and, if necessary, seek expert advice.

If level A alerts remain, which you believe to be justified deviations, and you intend to submit this CIF for publication in a journal, you should additionally insert an explanation in your CIF using the Validation Reply Form (VRF) below. This will allow your explanation to be considered as part of the review process.

Validation response form

Please find below a validation response form (VRF) that can be filled in and pasted into your CIF.

```
# start Validation Reply Form
_vrf_PUBL004_GLOBAL
;
PROBLEM: The contact author’s name and address are missing,
RESPONSE: ...
;
_vrf_PUBL005_GLOBAL
;
PROBLEM: _publ_contact_author_email, _publ_contact_author_fax and
RESPONSE: ...
;
_vrf_PUBL006_GLOBAL
;
PROBLEM: _publ_requested_journal is missing
RESPONSE: ...
;
_vrf_PUBL008_GLOBAL
;
PROBLEM: _publ_section_title is missing. Title of paper.
RESPONSE: ...
;
_vrf_PUBL009_GLOBAL
;
PROBLEM: _publ_author_name is missing. List of author(s) name(s).
RESPONSE: ...
;
_vrf_PUBL010_GLOBAL
;
PROBLEM: _publ_author_address is missing. Author(s) address(es).
RESPONSE: ...
;
_vrf_PUBL012_GLOBAL
;
PROBLEM: _publ_section_abstract is missing.
RESPONSE: ...
;
# end Validation Reply Form
```

If you wish to submit your CIF for publication in Acta Crystallographica Section C or E, you should upload your CIF via the web. If you wish to submit your CIF for publication in IUCrData you should upload your CIF via the web. If your CIF is to form part of a submission to another IUCr journal, you will be asked, either during electronic submission or by the Co-editor handling your paper, to upload your CIF via our web site.

PLATON version of 22/03/2021; check.def file version of 19/03/2021

Datablock I - ellipsoid plot

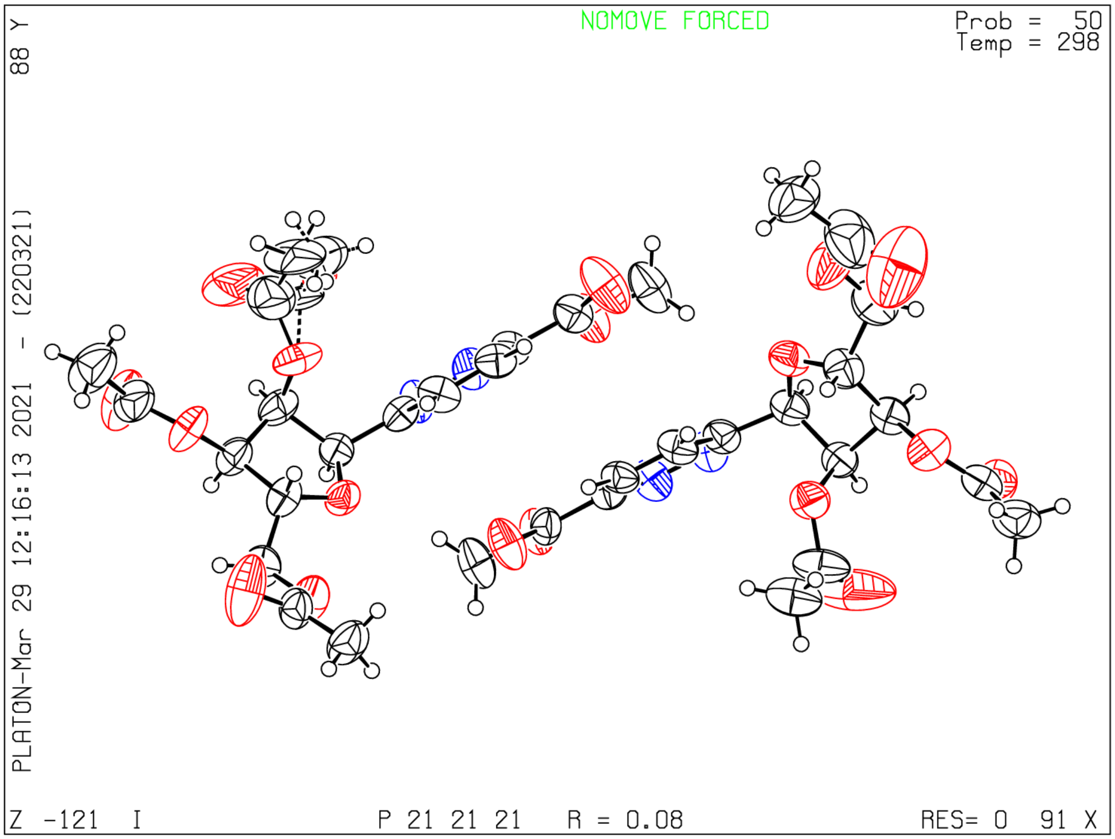

Supplement: Supplementary file 1 [file molecules-26-02341-s001.zip › molecules-1189220-supplementary/molecules-1189220-SI/checkcif.pdf]
