# Supplementary material for: A One-Pot Approach to Novel Pyridazine C-Nucleosides
Source: Molecules. 2021 Apr 17;26(8):2341. doi: 10.3390/molecules26082341 (PMC8074166; doi:10.3390/molecules26082341)
Supplement: Supplementary file 1 [file molecules-26-02341-s001.zip › molecules-1189220-supplementary/molecules-1189220-SI/Supplementary Material.pdf]

A One-pot Approach to Novel Pyridazine *C*-Nucleosides

Flavio Cermola [1,*], Serena Vella [2], Marina DellaGreca [1], Angela Tuzi [1] and Maria Rosaria Iesce [1]

[1] Dipartimento di Scienze Chimiche, Università di Napoli Federico II, Complesso Universitario di M. Sant'Angelo, Via Cintia, 80126 - Napoli, Italy; dellagre@unina.it, angela.tuzi@unina.it, iesce@unina.it
[2] Erbagil s.r.l. Via L. Settembrini, 13 82037 - Telese Terme (BN), Italy; s.vella@erbagil.com
* Correspondence: cermola@unina.it

Supplementary Materials

Table of contents

^{1}H NMR ($CDCl_3$) of ribofuranosyl furan **2a**. S1
^{13}C NMR ($CDCl_3$) of ribofuranosyl furan **2a**. S2
^{1}H NMR ($CDCl_3$) of ribofuranosyl furan **2b**. S3
^{13}C NMR ($CDCl_3$) of ribofuranosyl furan **2b**. S4
^{1}H NMR (C_6D_6) of ribofuranosyl furan **2c**. S5
COSY (C_6D_6)of ribofuranosyl furan **2c**. S6
NOESY (C_6D_6) of ribofuranosyl furan **2c**. S7
^{1}H NMR ($CDCl_3$) of furan **6d**. S8
^{13}C NMR ($CDCl_3$) of furan **6d**. S9
^{1}H NMR ($CDCl_3$) of pyridazine **5a**. S10
^{13}C NMR ($CDCl_3$) of pyridazine **5a**. S11
COSY ($CDCl_3$) of pyridazine **5a**. S12
Simmetrized COSY ($CDCl_3$) of pyridazine **5a**. S13
NOESY ($CDCl_3$) of pyridazine **5a**. S14
^{1}H NMR ($CDCl_3$) of pyridazine **5b**. S15
^{13}C NMR ($CDCl_3$) of pyridazine **5b**. S16
COSY ($CDCl_3$) of pyridazine **5b**. S17
NOESY ($CDCl_3$) of pyridazine **5b**. S18
^{1}H NMR (DMSO) of pyridazine **5c**. S19
Expanded ^{1}H NMR (DMSO) of pyridazine **5c**. S20
Expanded ^{1}H NMR (DMSO) of pyridazine **5c**. S21
^{13}C NMR (DMSO) of pyridazine **5c**. S22
COSY (DMSO) of pyridazine **5c**. S23
Expanded COSY (DMSO) of pyridazine **5c**. S24
^{1}H NMR ($CDCl_3$) of pyridazine **5d**. S25
^{13}C NMR ($CDCl_3$) of pyridazine **5d**. S26
COSY ($CDCl_3$) of pyridazine **5d**. S27
Expanded COSY ($CDCl_3$) of pyridazine **5d**. S28
NOESY ($CDCl_3$) of pyridazine **5d**. S29
Expanded NOESY ($CDCl_3$) of pyridazine **5d**. S30
X-ray diffraction analysis of compound **5d**. S31

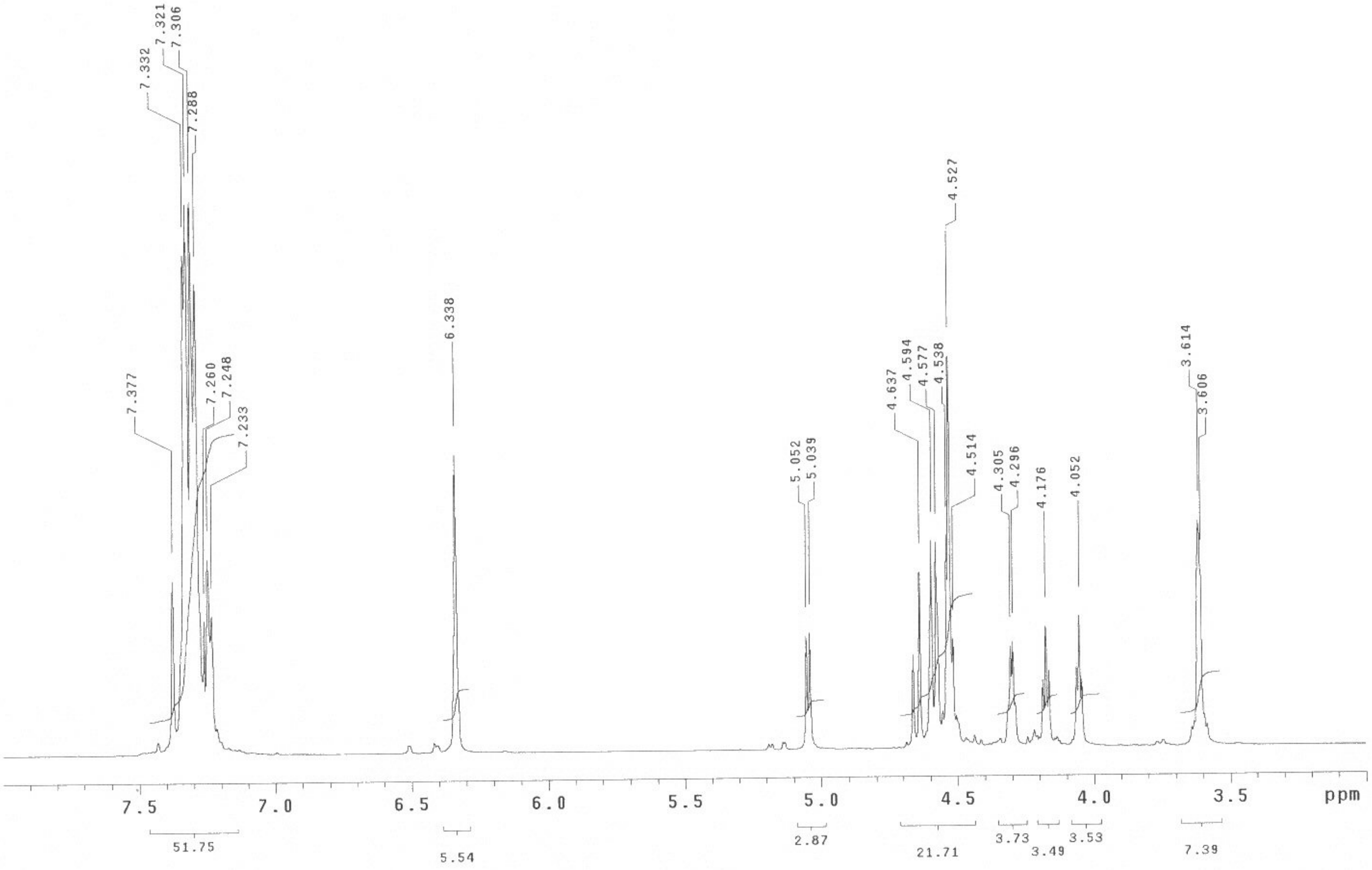

^{1}H NMR ($CDCl_3$) of ribofuranosyl furan 2a.

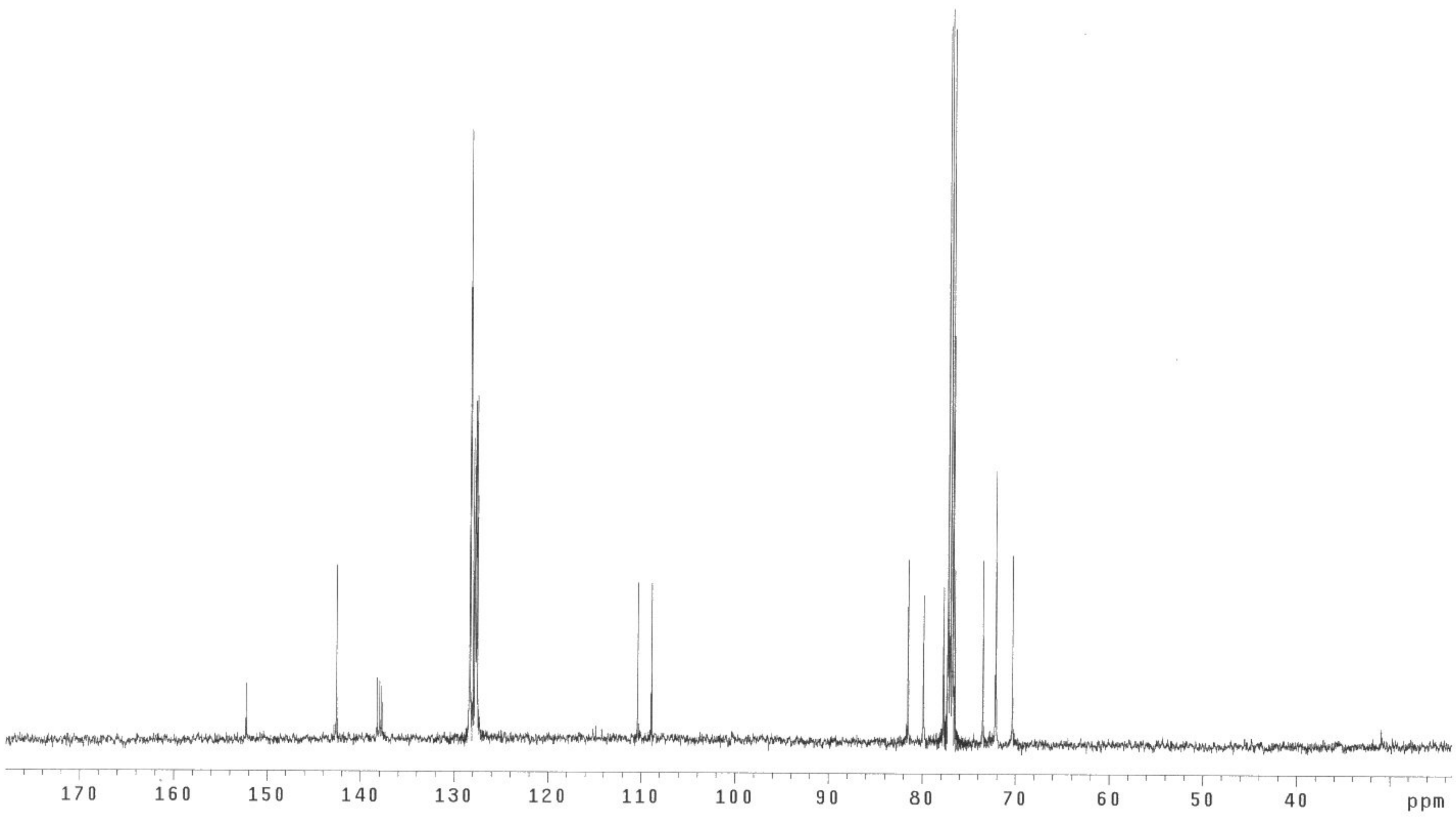

^{13}C NMR ($CDCl_3$) of ribofuranosyl furan 2a.

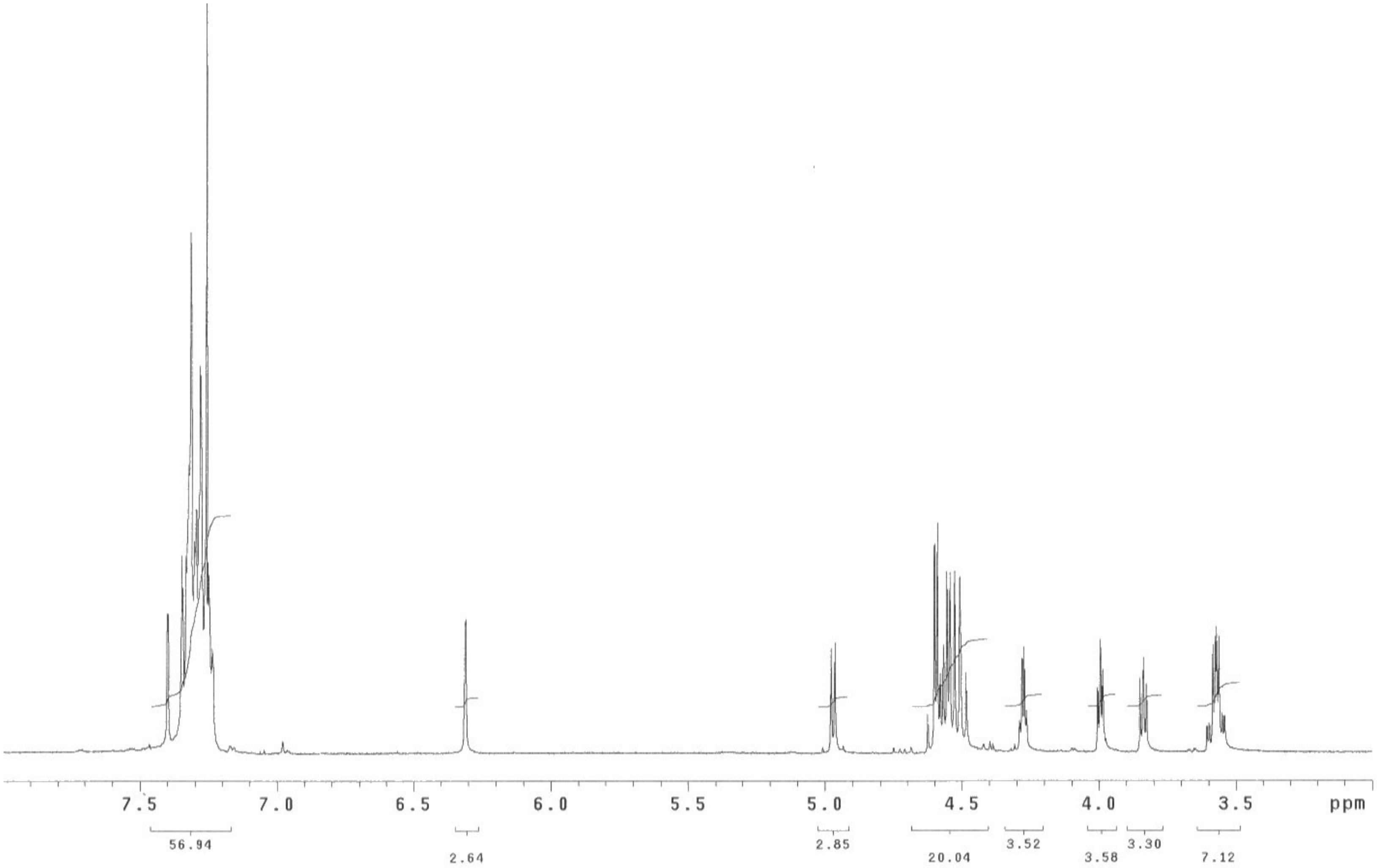

^{1}H NMR ($CDCl_3$) of ribofuranosyl furan 2b.

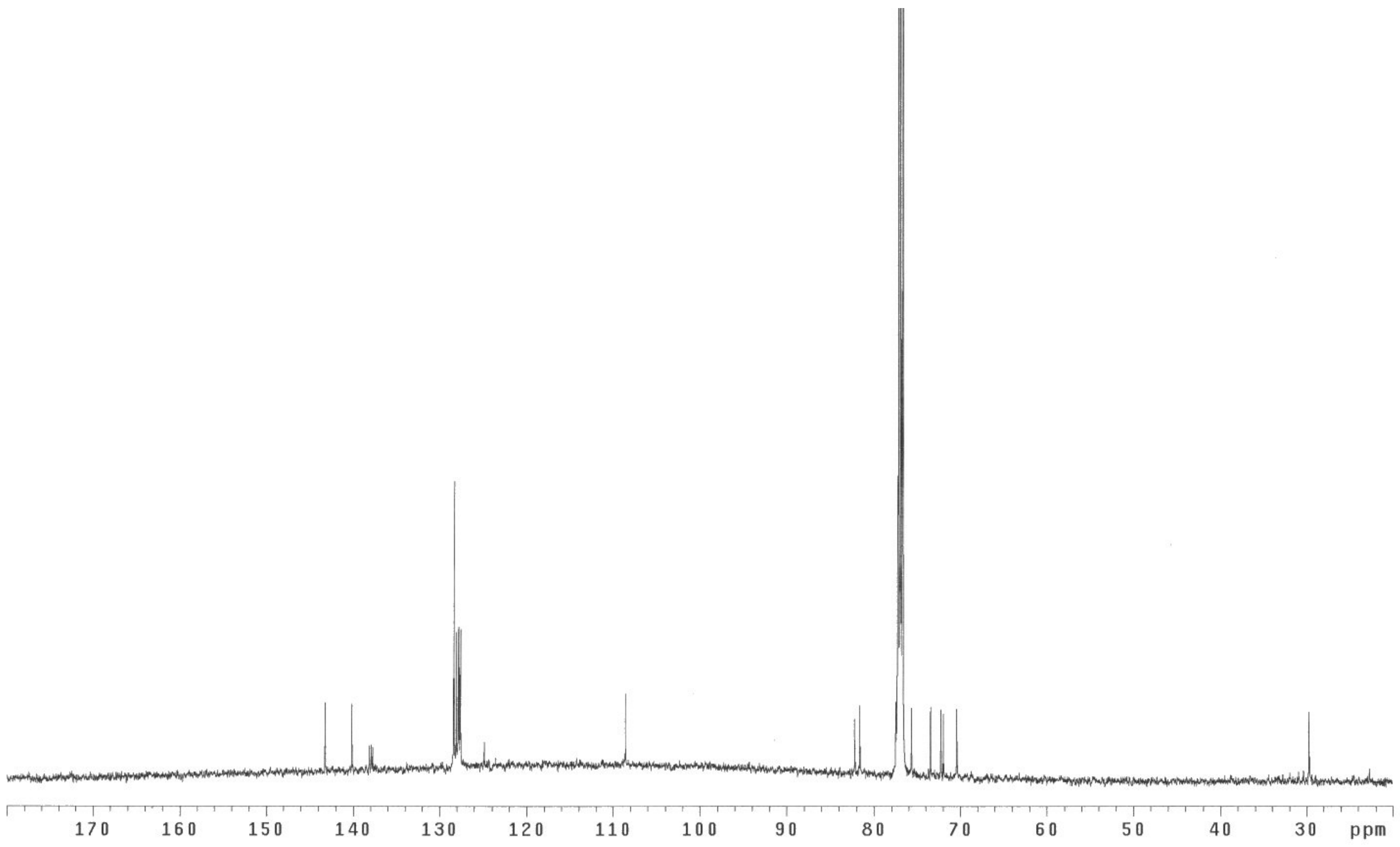

^{13}C NMR ($CDCl_3$) of ribofuranosyl furan 2b.

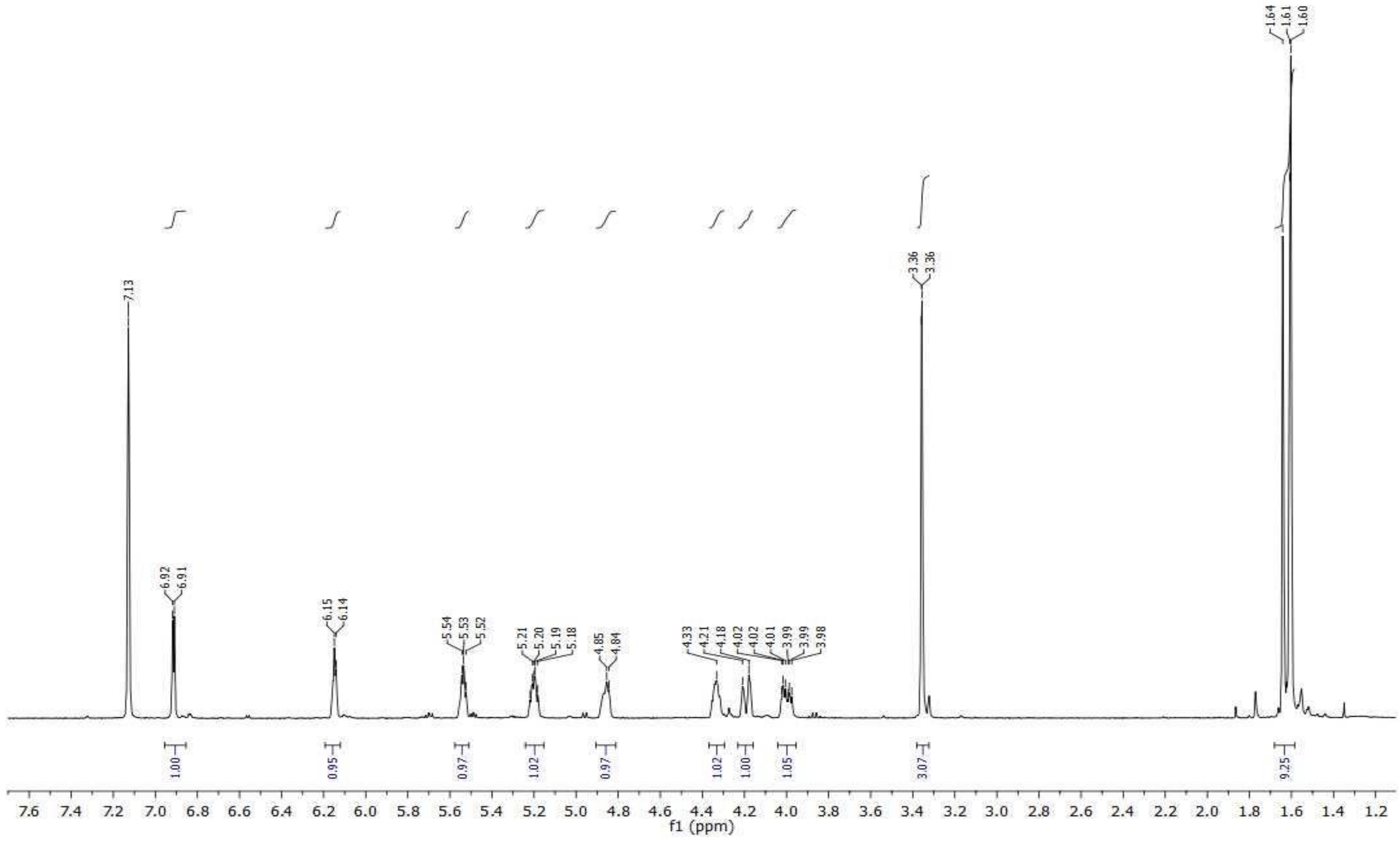

^{1}H NMR (C_6D_6) of ribofuranosyl furan 2c.

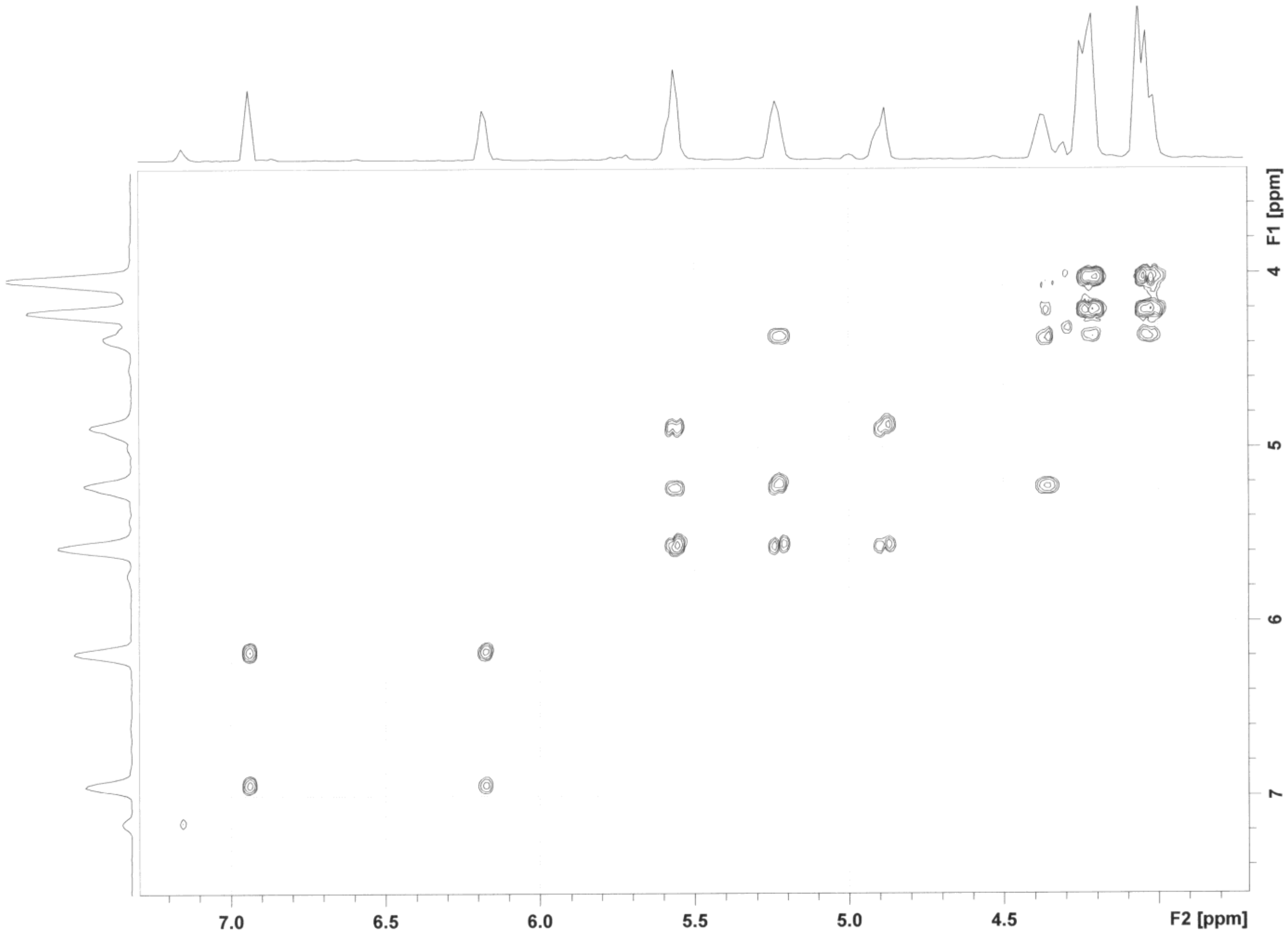

COSY (C_6D_6) of ribofuranosyl furan 2c.

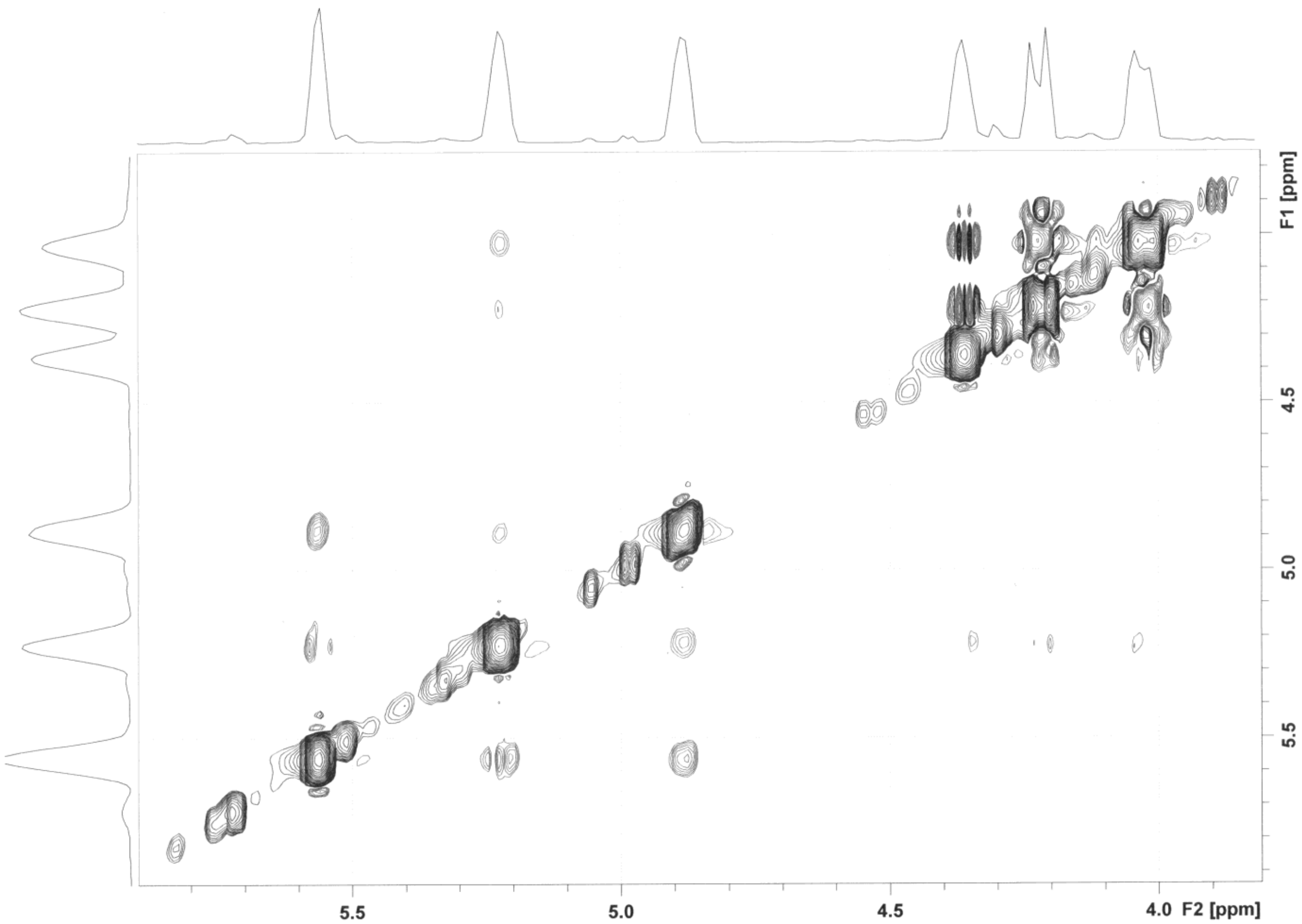

NOESY (C_6D_6) of ribofuranosyl furan 2c.

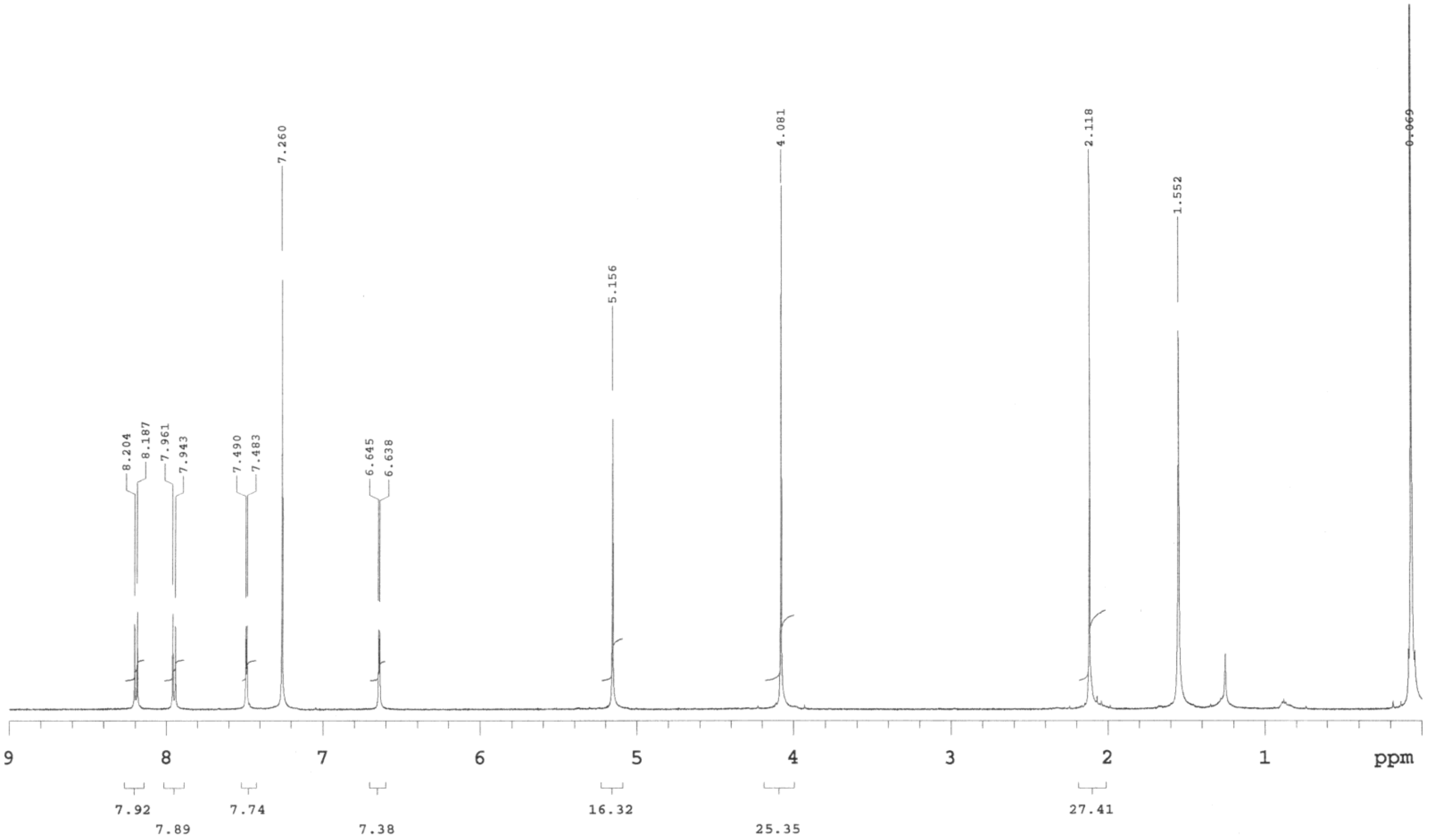

^{1}H NMR ($CDCl_3$) of furan 6d.

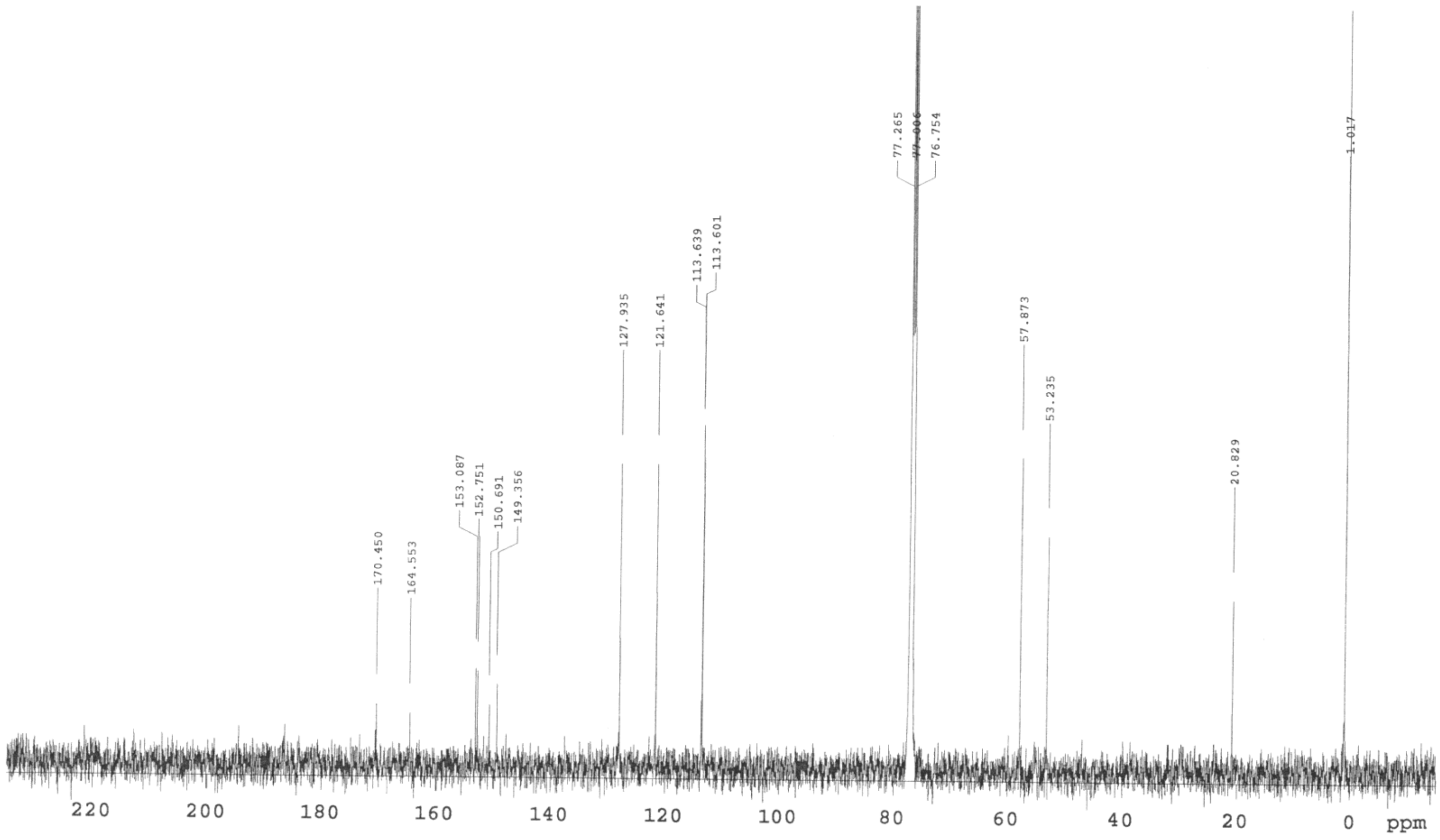

^{13}C NMR ($CDCl_3$) of furan 6d.

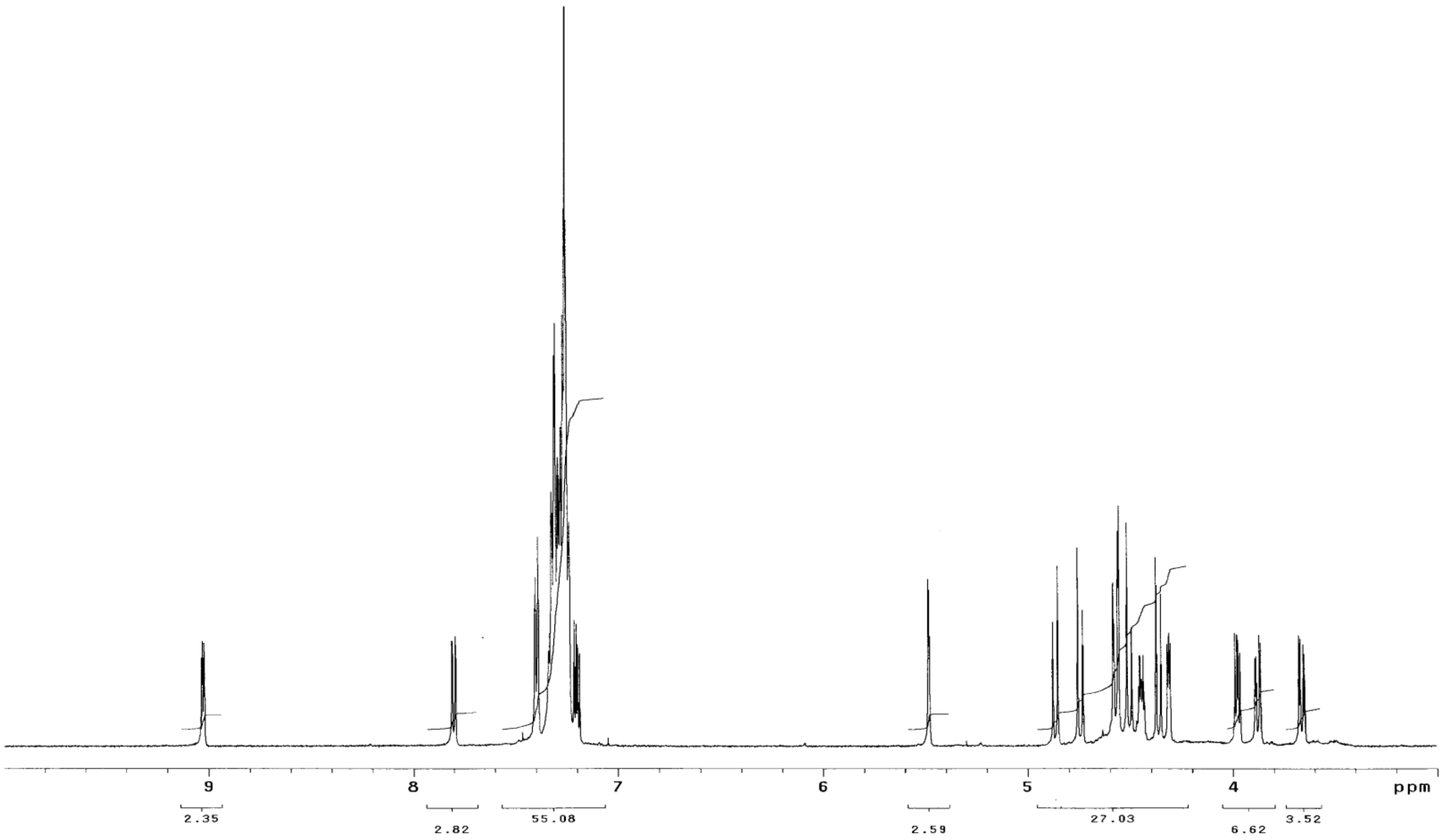

^{1}H NMR ($CDCl_3$) of pyridazine 5a.

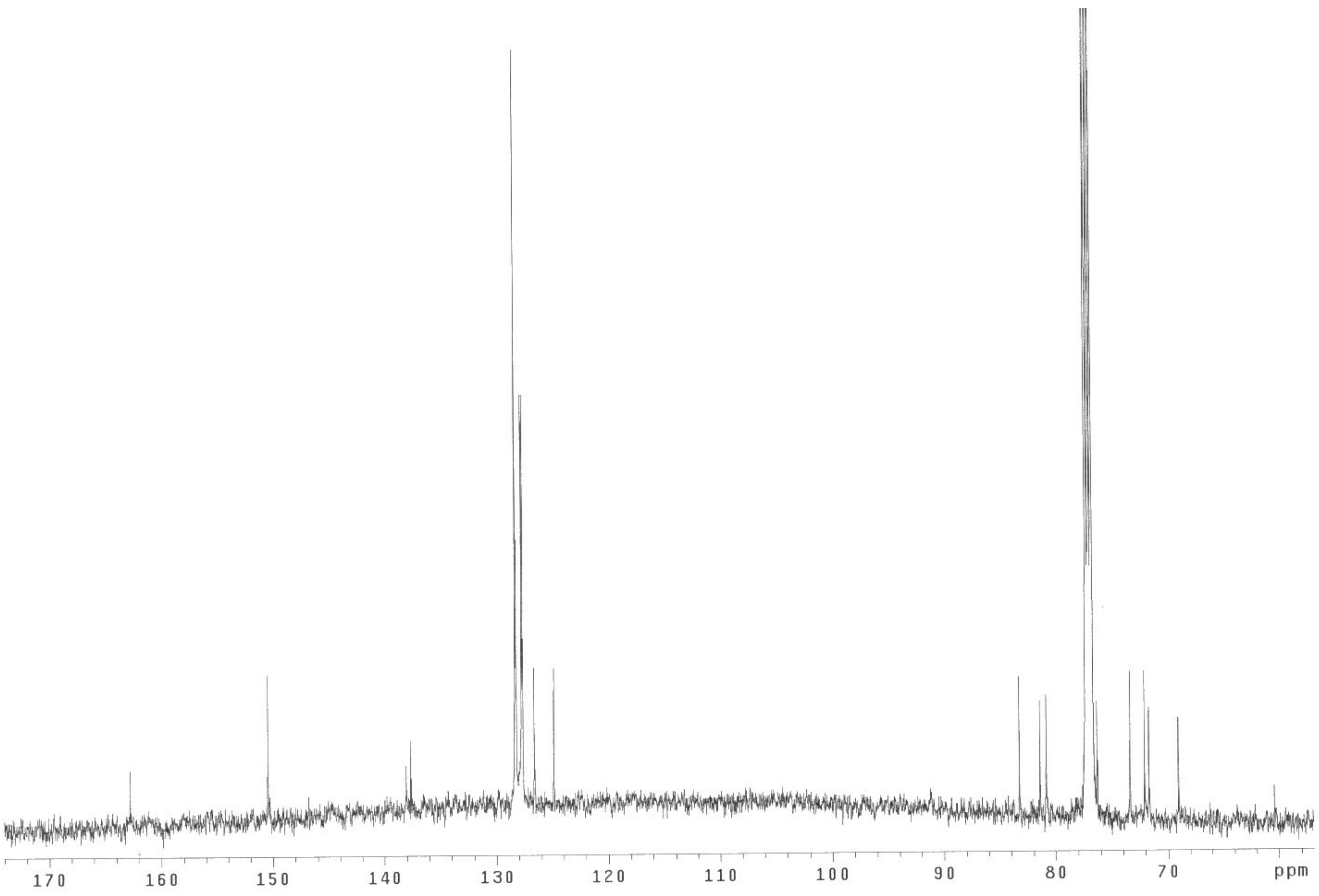

^{13}C NMR ($CDCl_3$) of pyridazine **5a**.

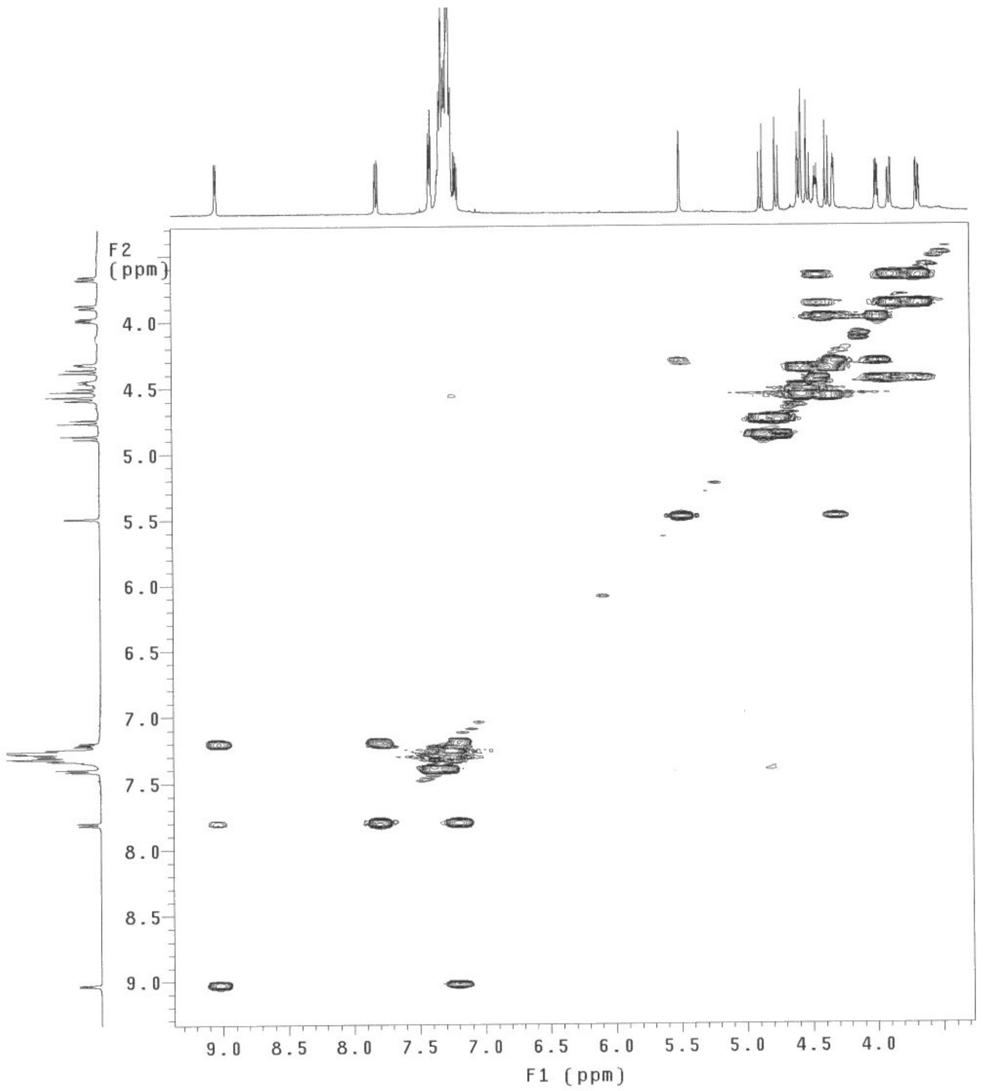

COSY ($CDCl_3$) of pyridazine 5a.

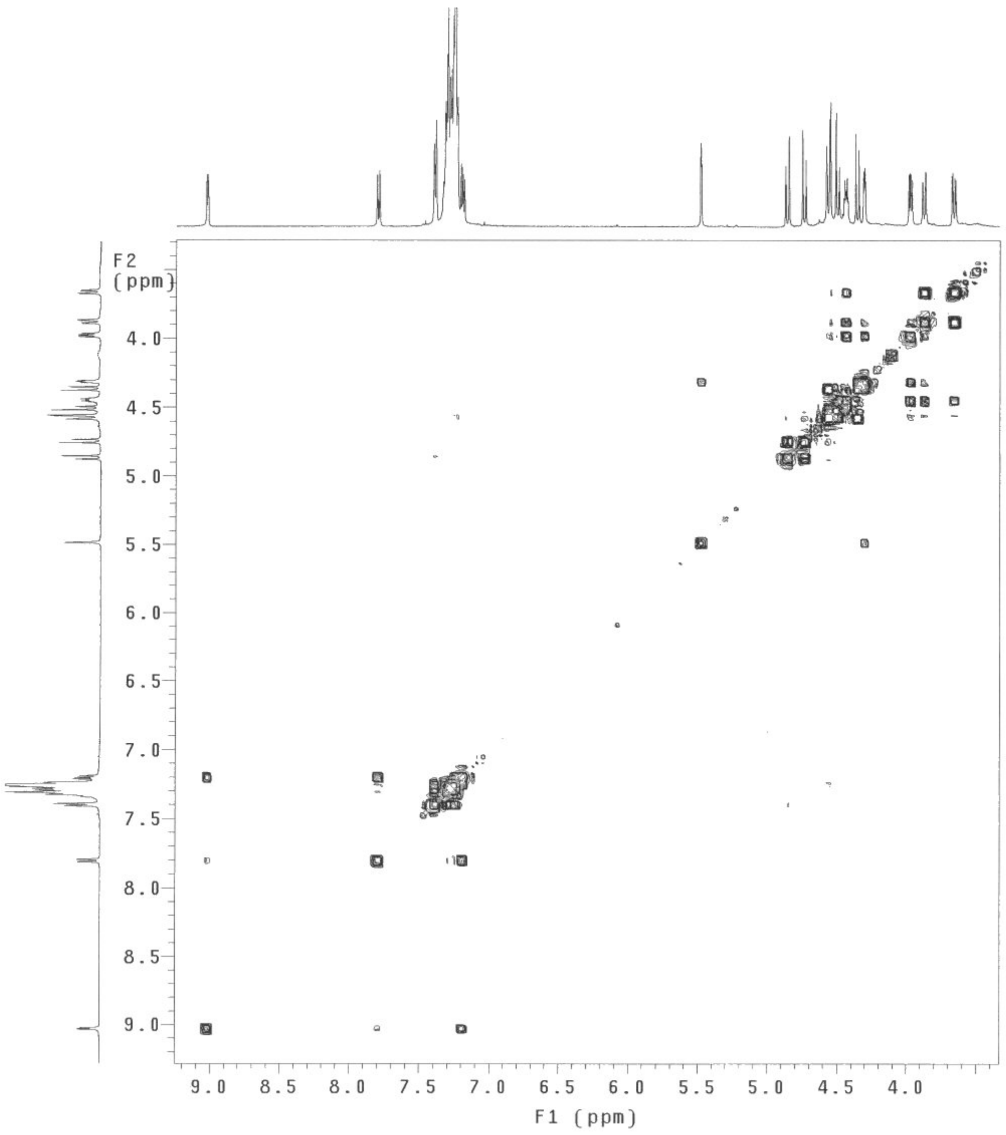

Symmetrized COSY ($CDCl_3$) of pyridazine 5a.

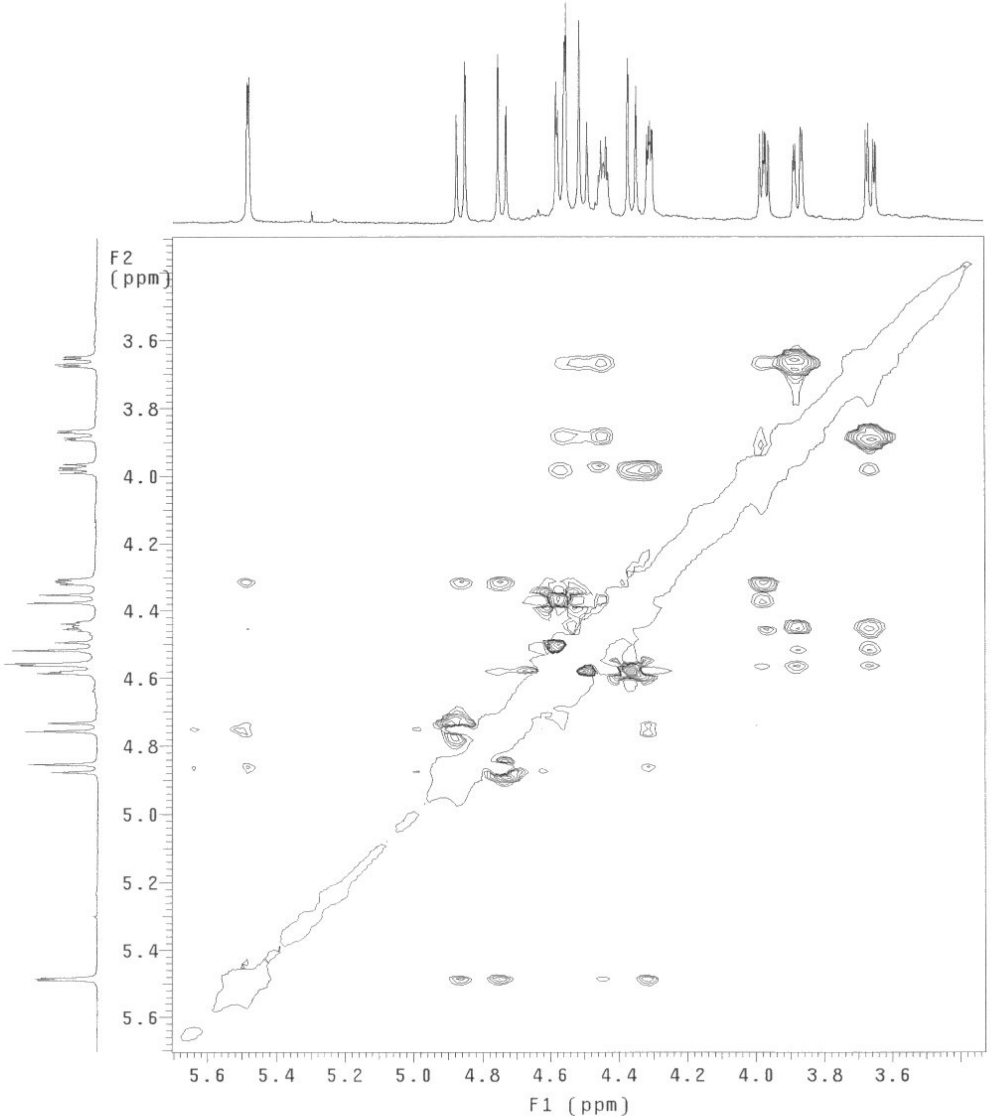

NOESY ($CDCl_3$) of pyridazine 5a.

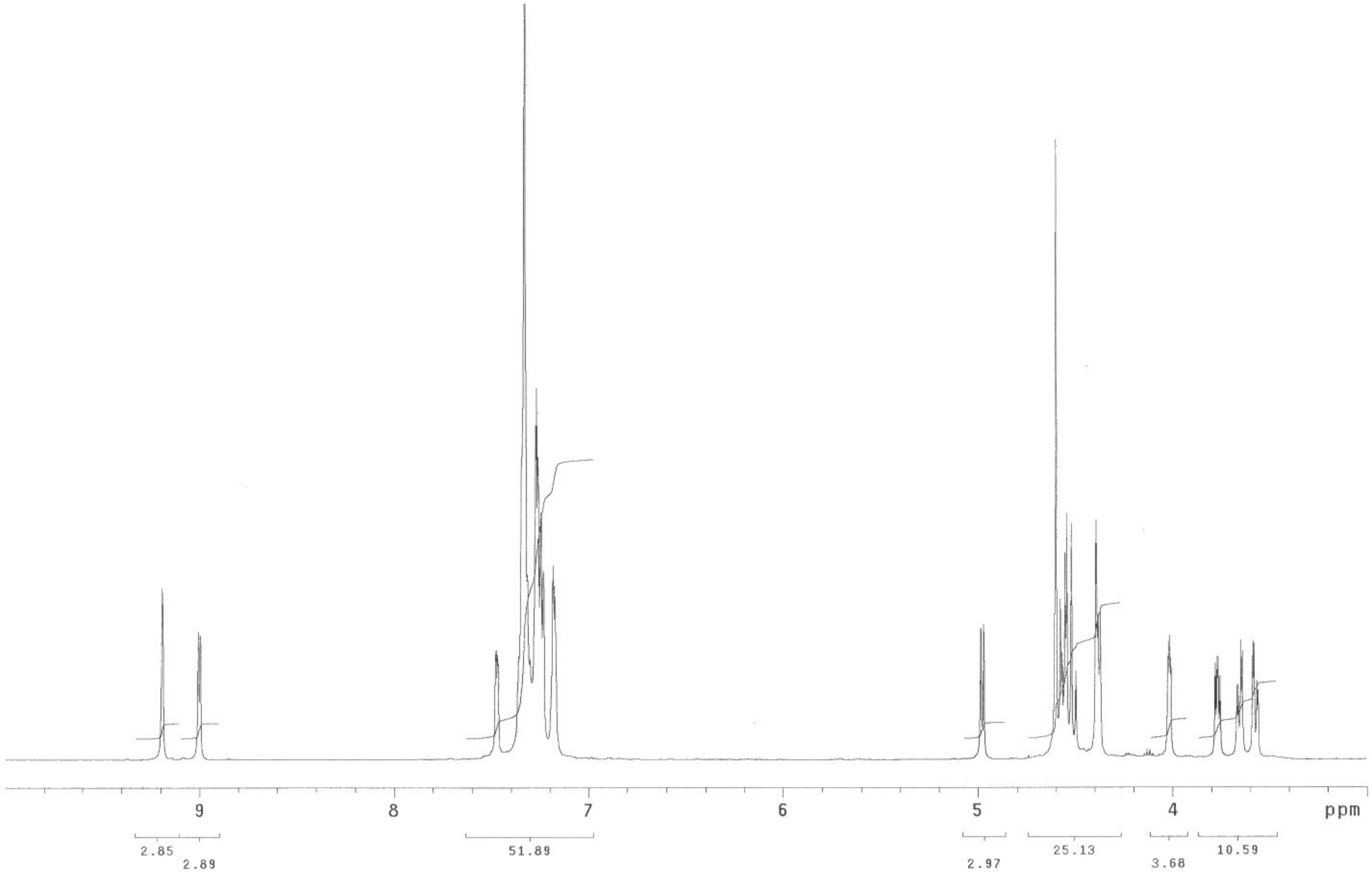

^{1}H NMR ($CDCl_3$) of pyridazine **5b**.

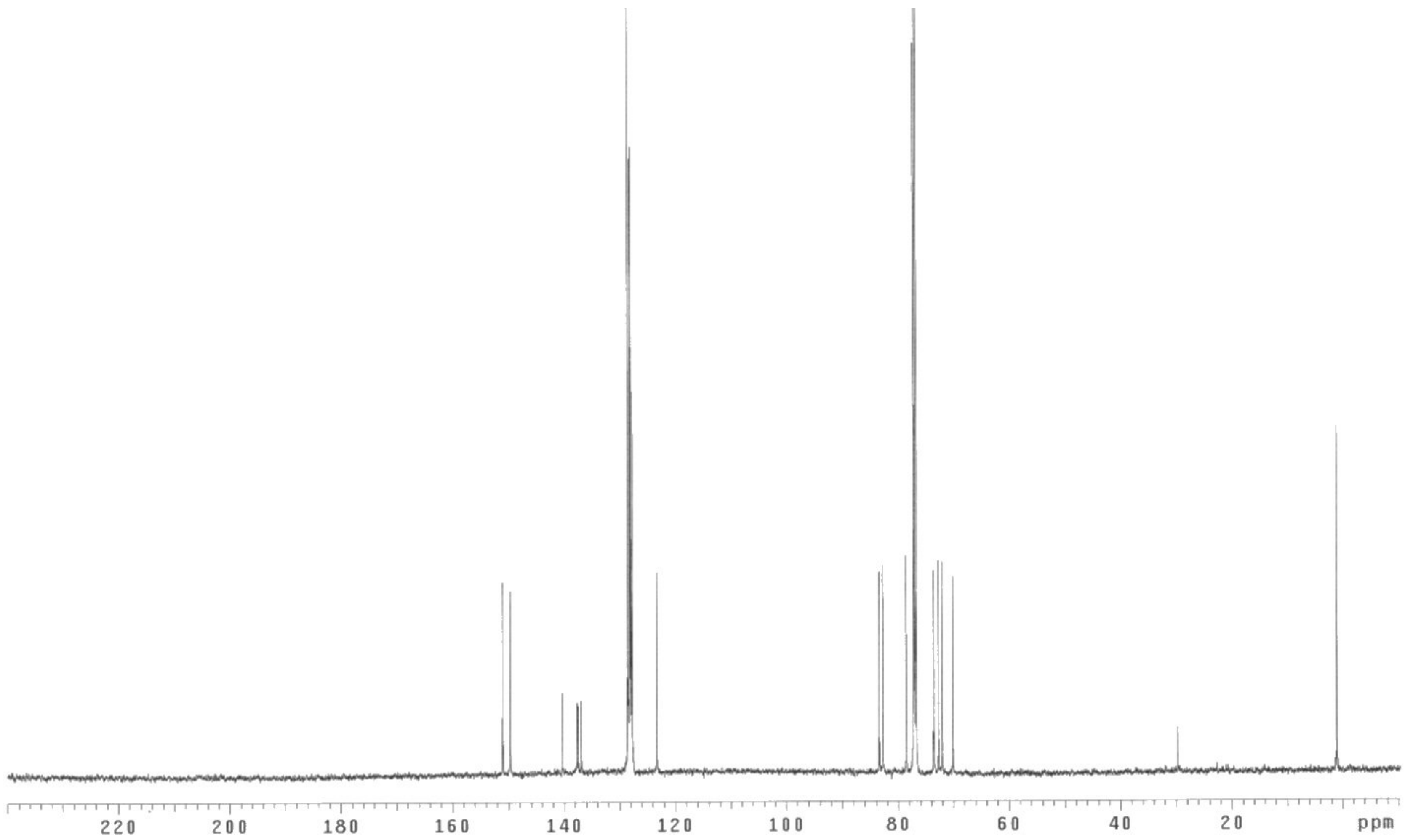

^{13}C NMR ($CDCl_3$) of pyridazine 5b.

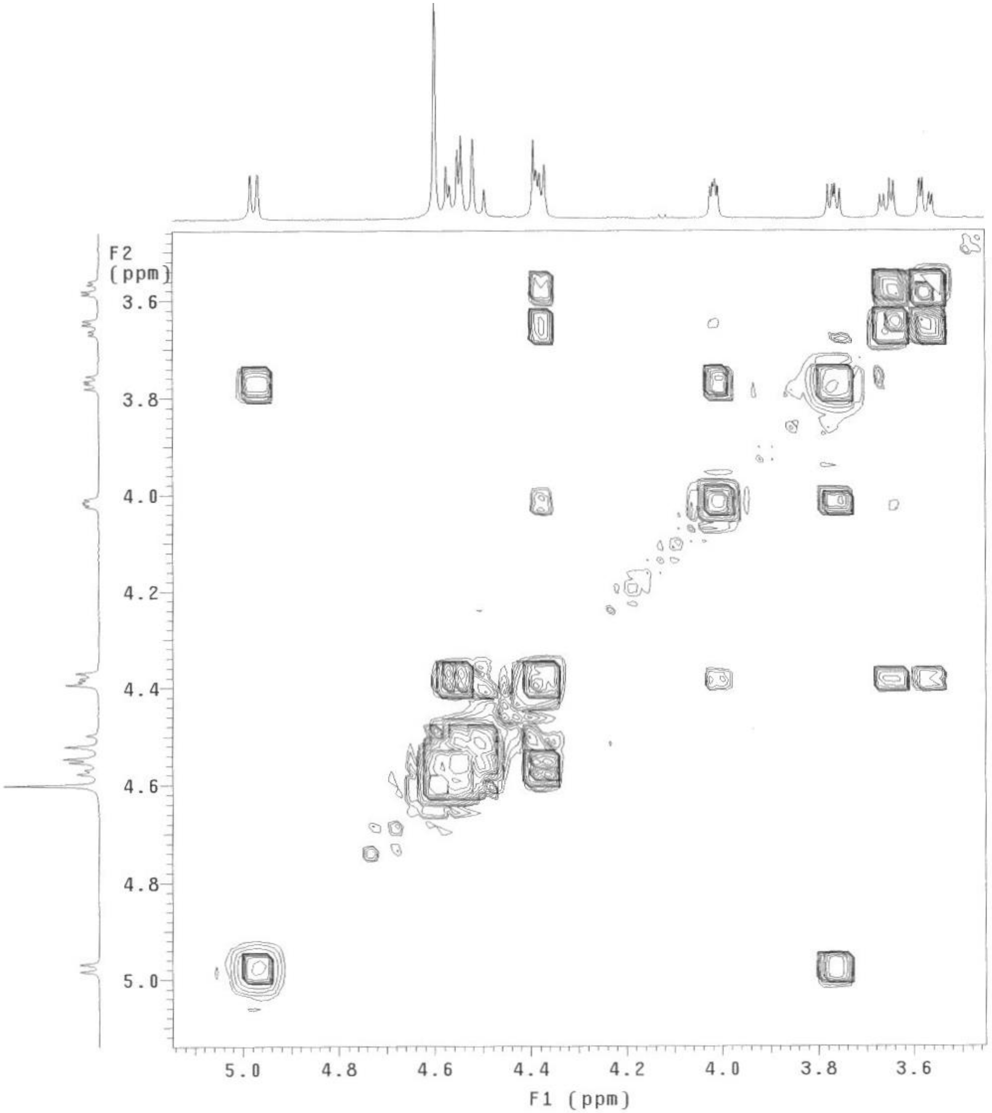

Symmetrized COSY ($CDCl_3$) of pyridazine 5b.

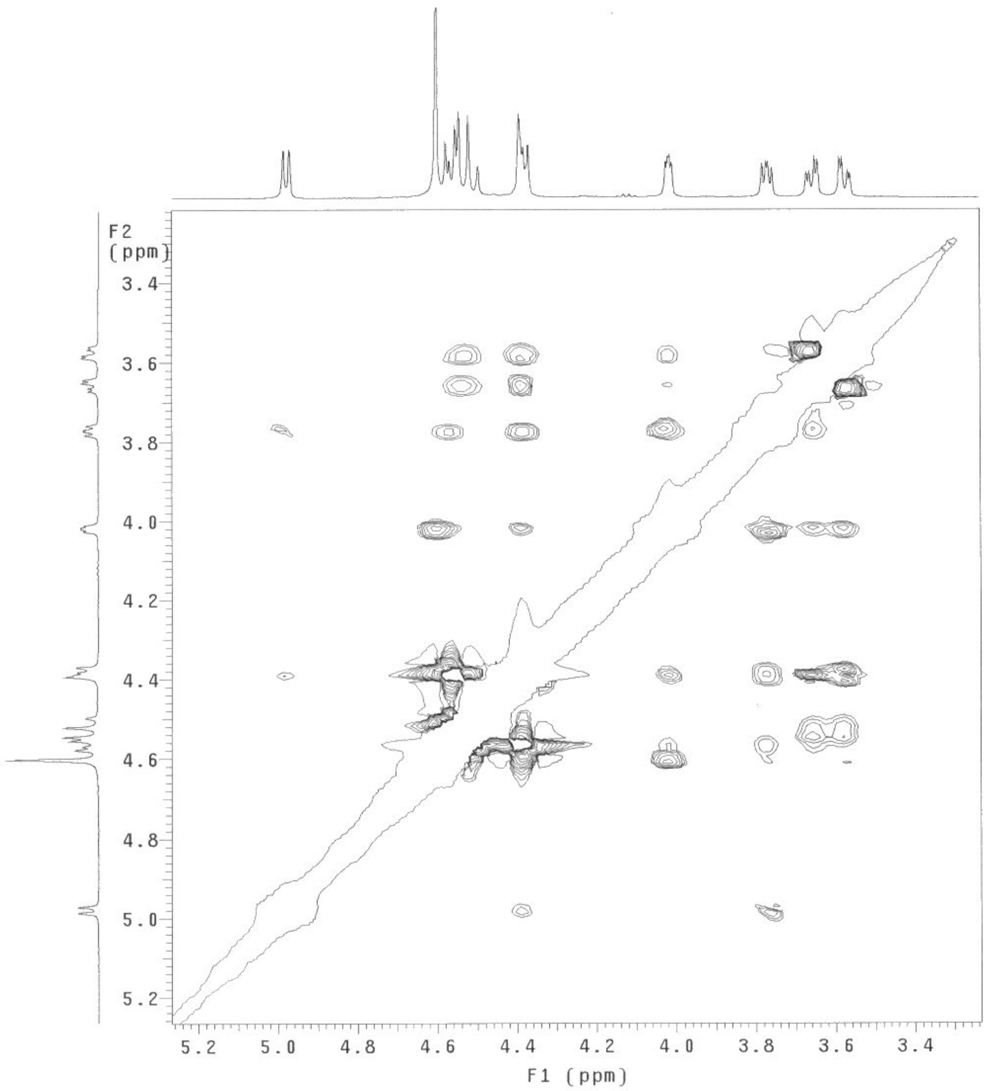

NOESY ($CDCl_3$) of pyridazine 5b.

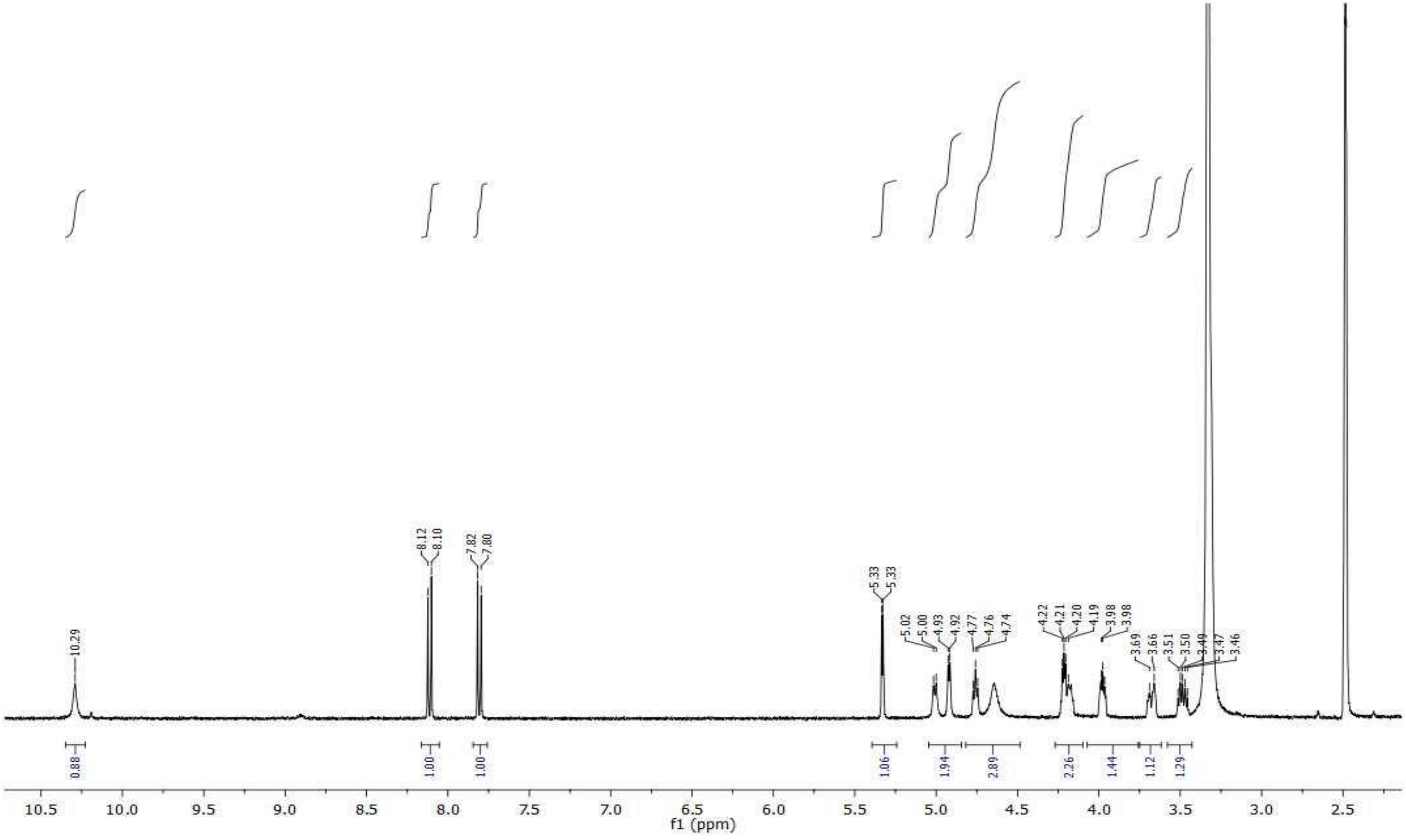

¹H NMR (DMSO) of pyridazine 5c.

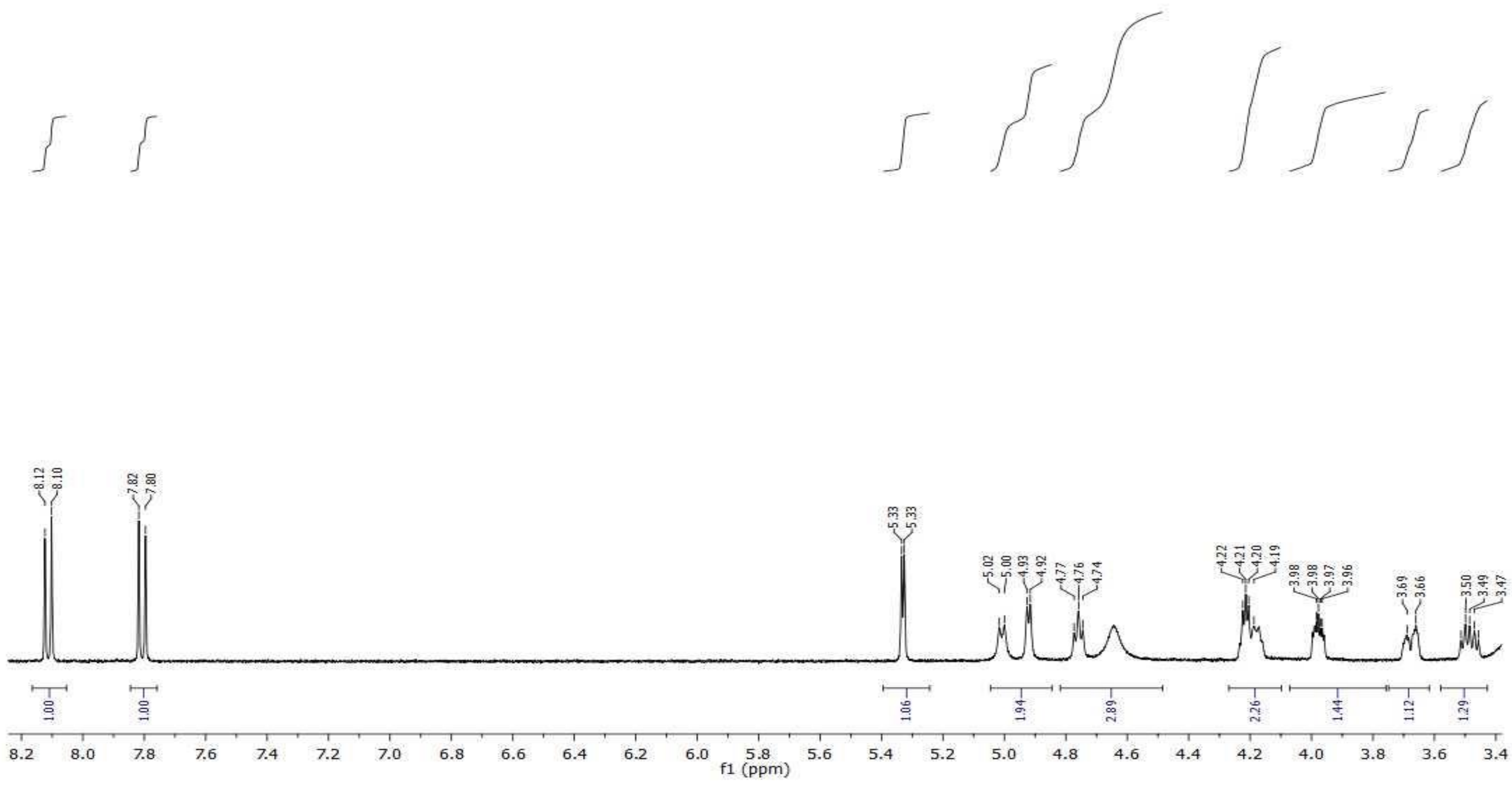

Expanded ^{1}H NMR (DMSO) of pyridazine 5c.

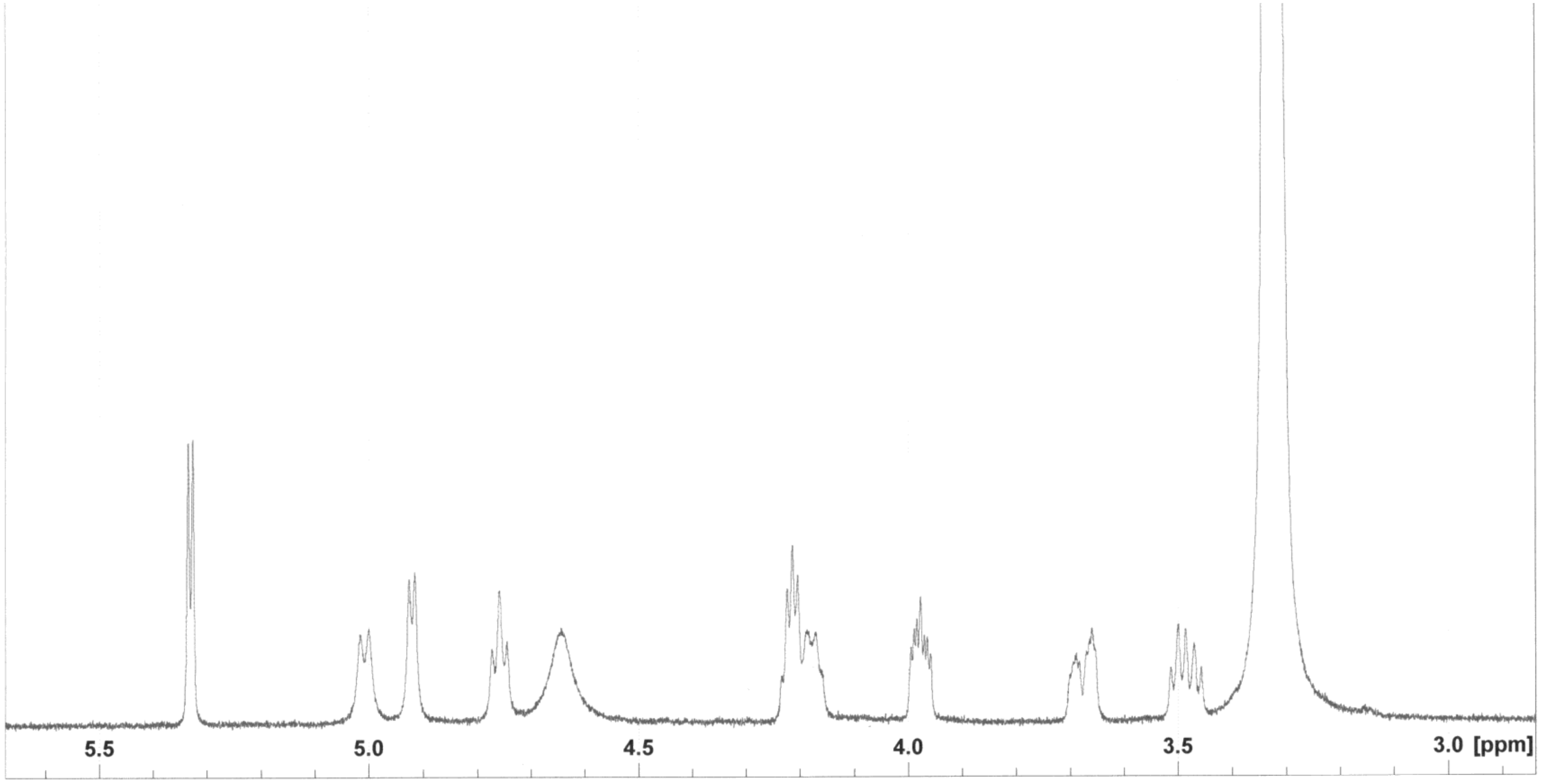

Expanded ^{1}H NMR (DMSO) of pyridazine 5c.

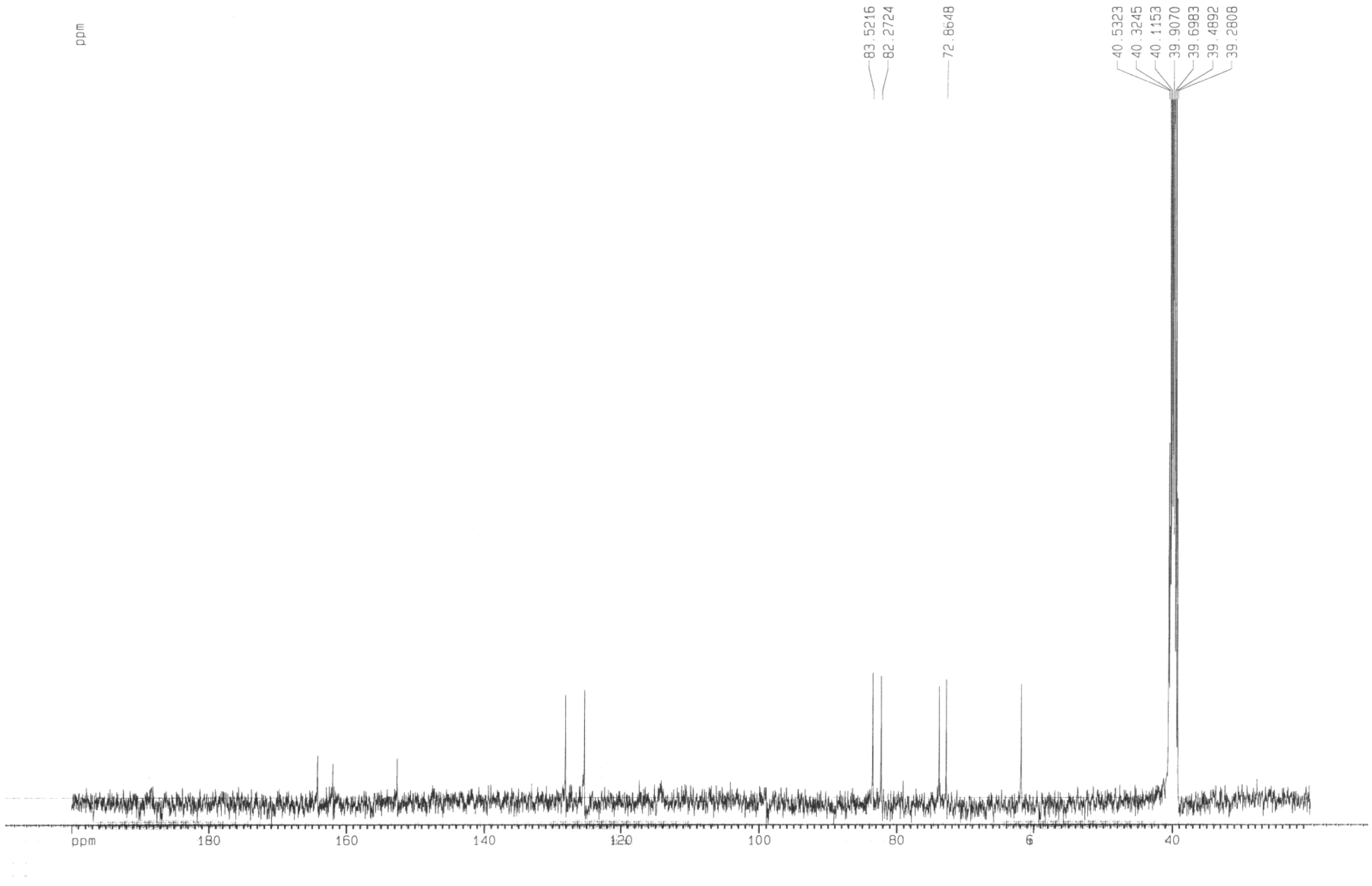

^{13}C NMR (DMSO) of pyridazine 5c.

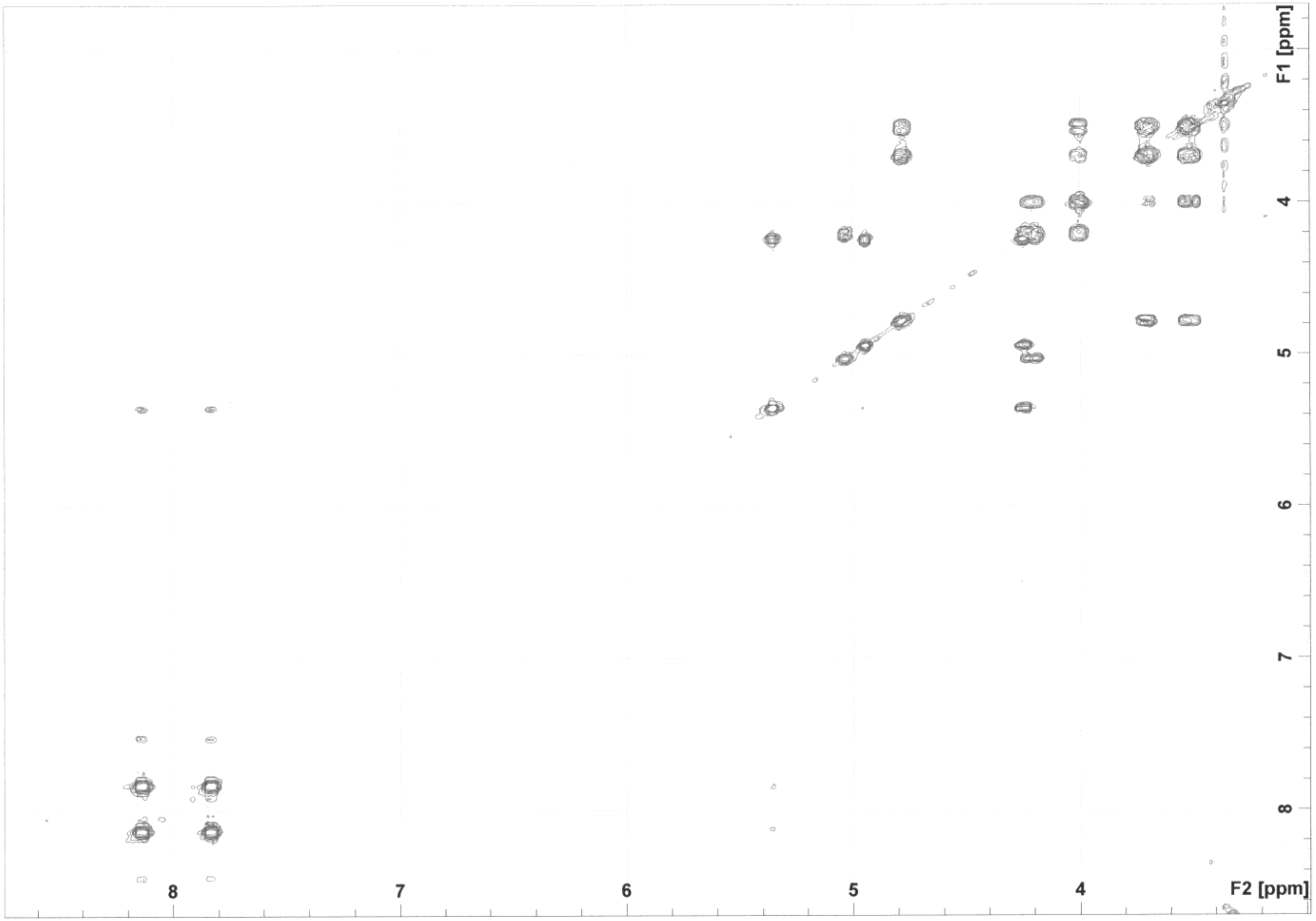

COSY (DMSO) of pyridazine 5c.

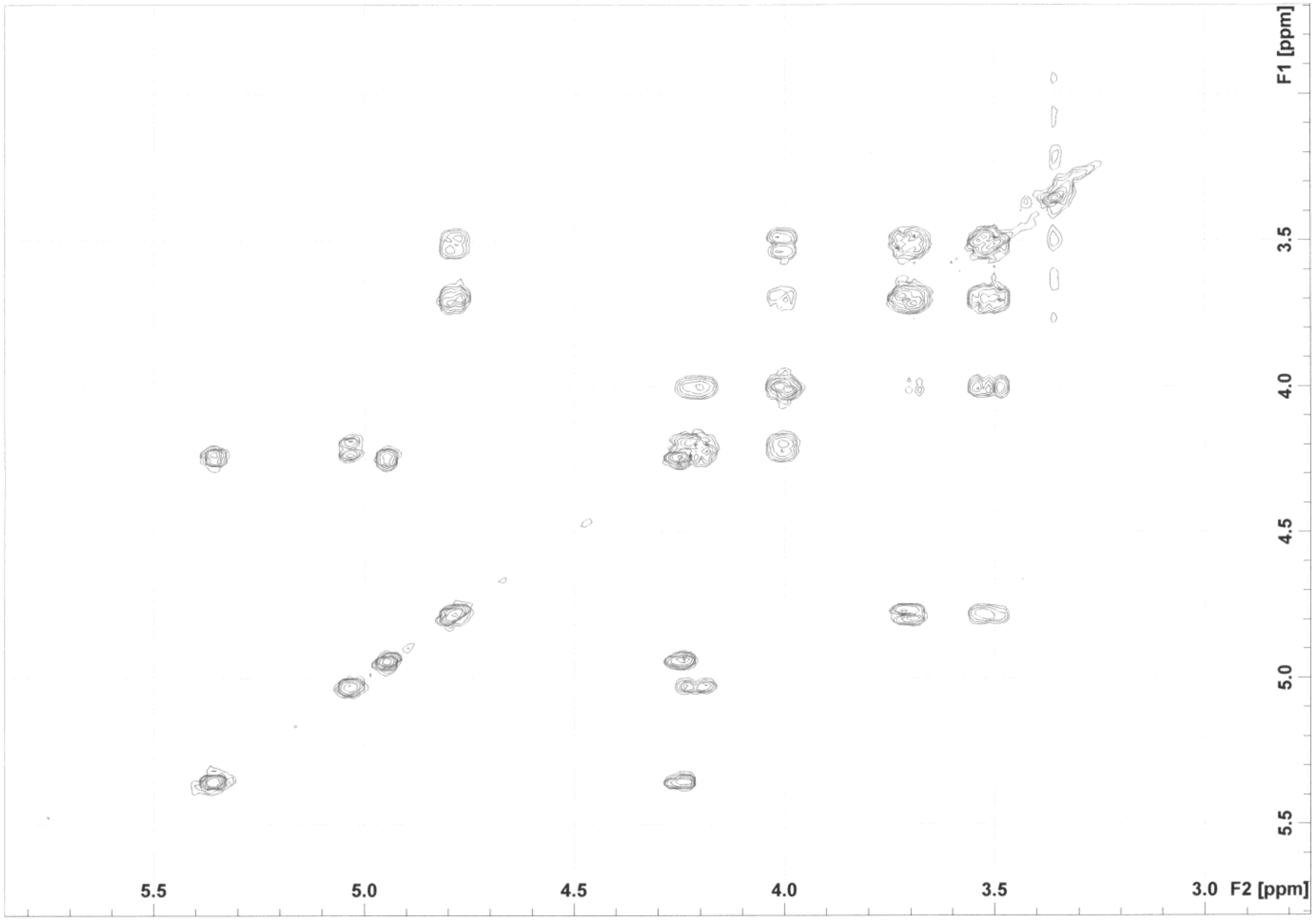

Expanded COSY (DMSO) of pyridazine 5c.

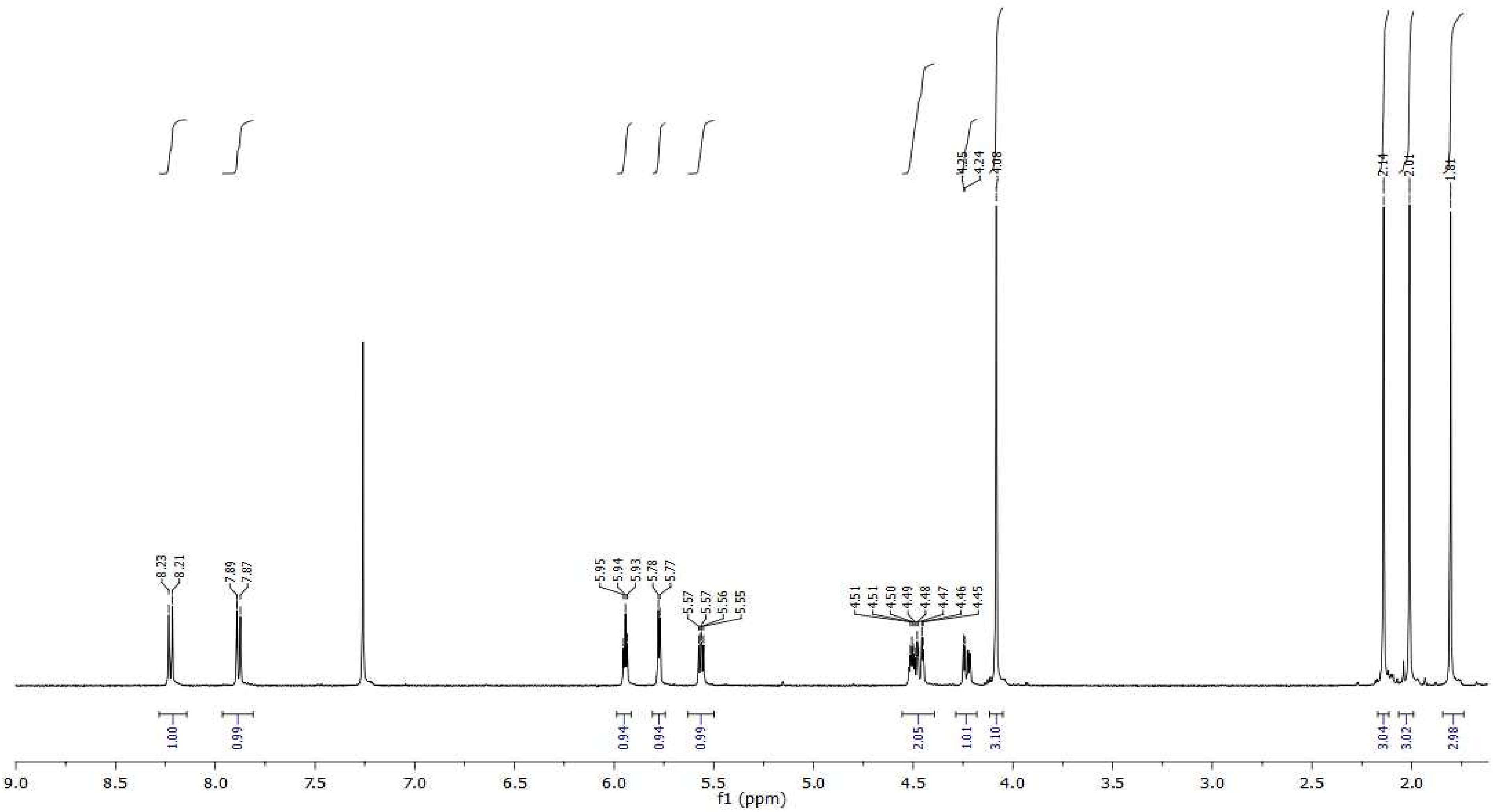

^{1}H NMR ($CDCl_3$) of pyridazine 5d.

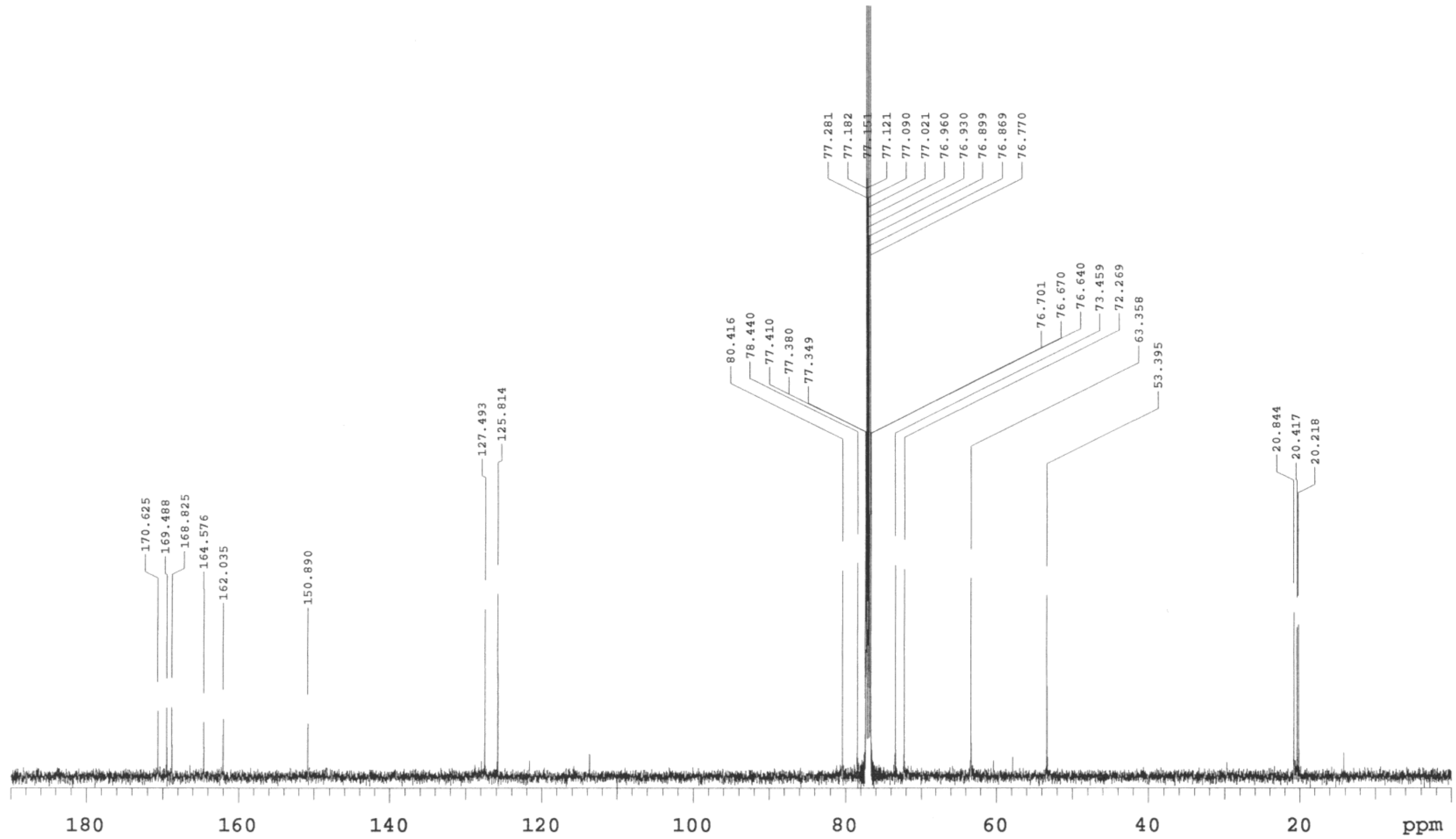

^{13}C NMR ($CDCl_3$) of pyridazine 5d.

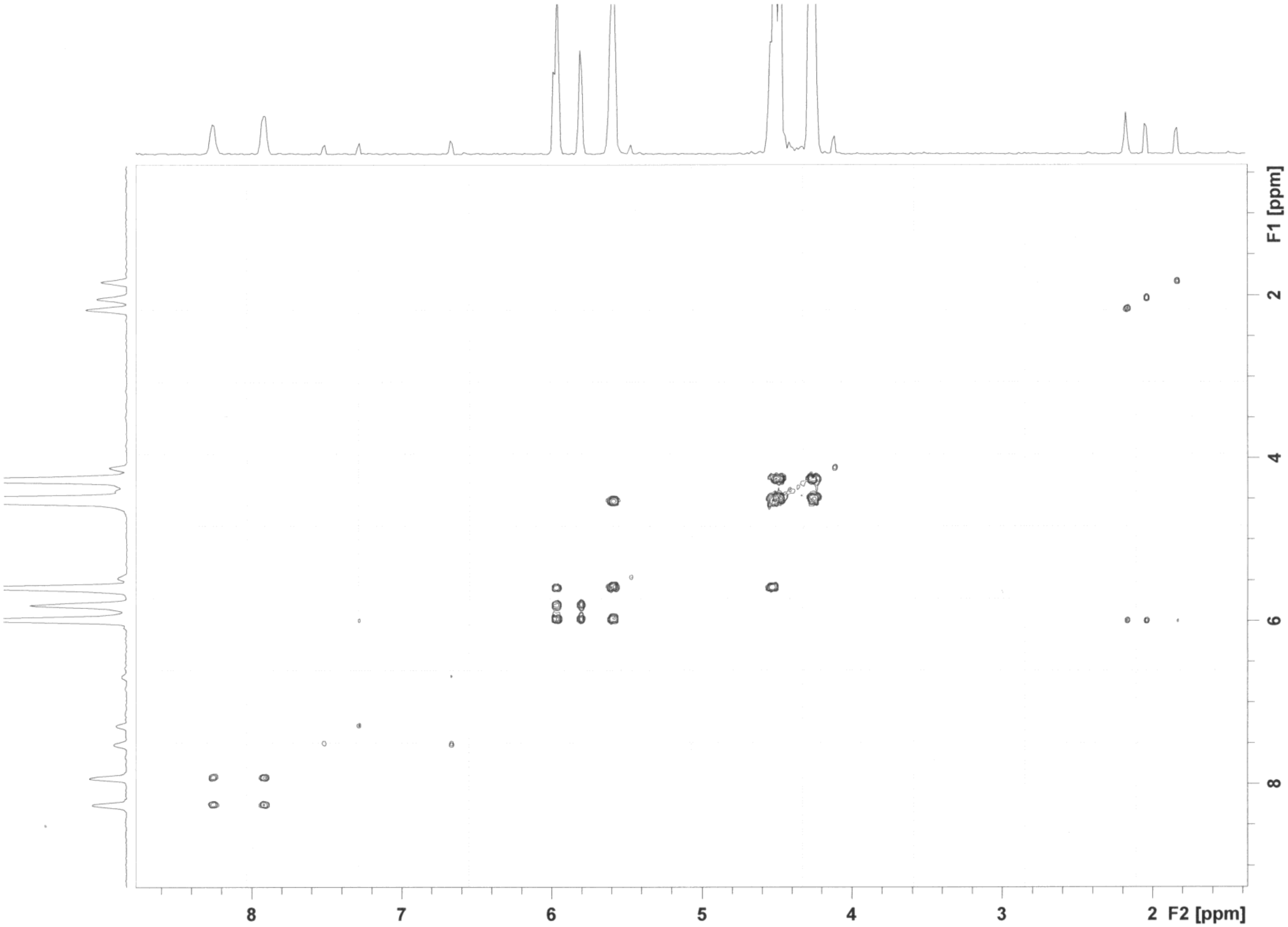

COSY (CDCl$_3$) of pyridazine 5d.

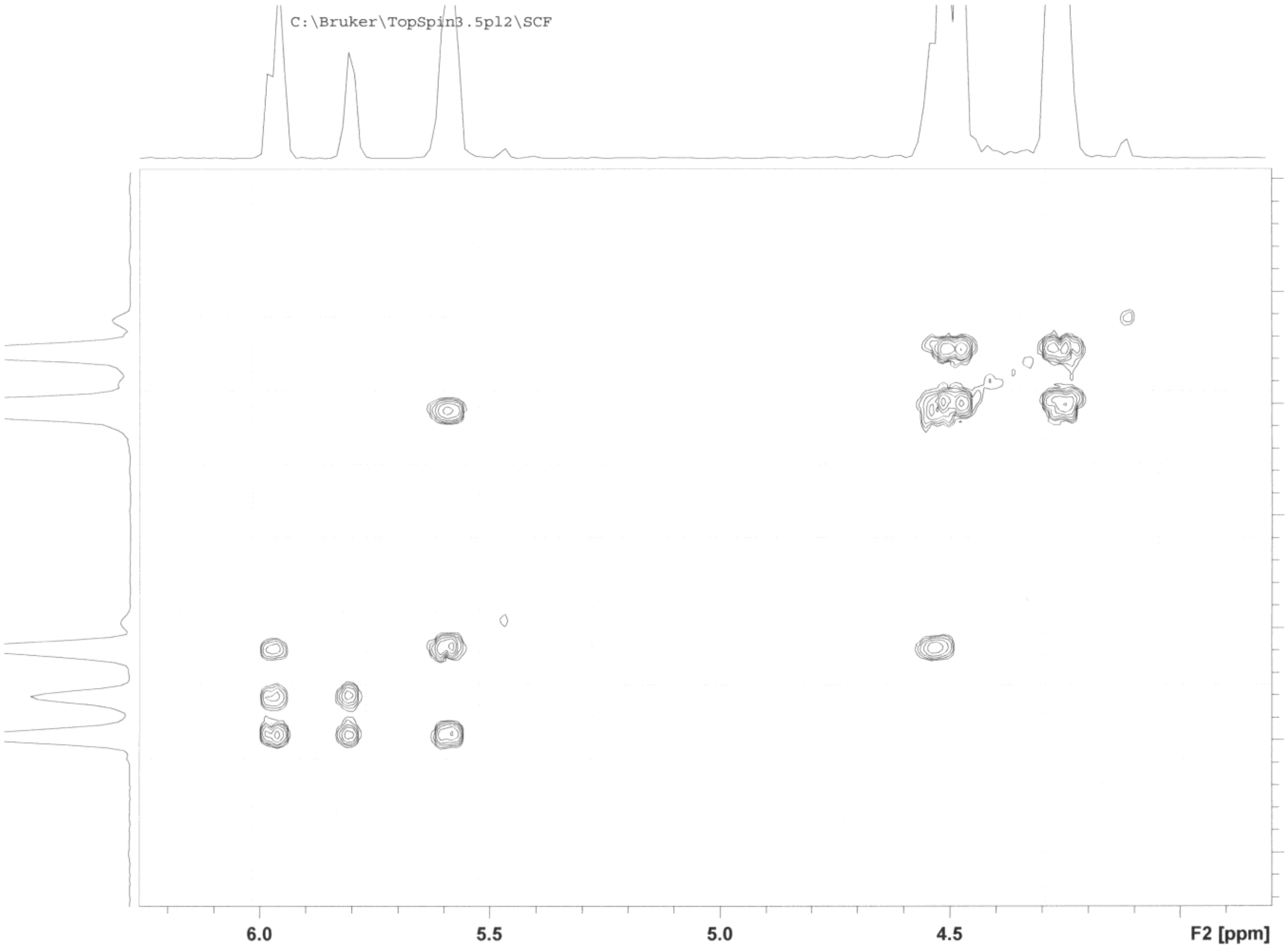

Expanded COSY ($CDCl_3$) of pyridazine 5d.

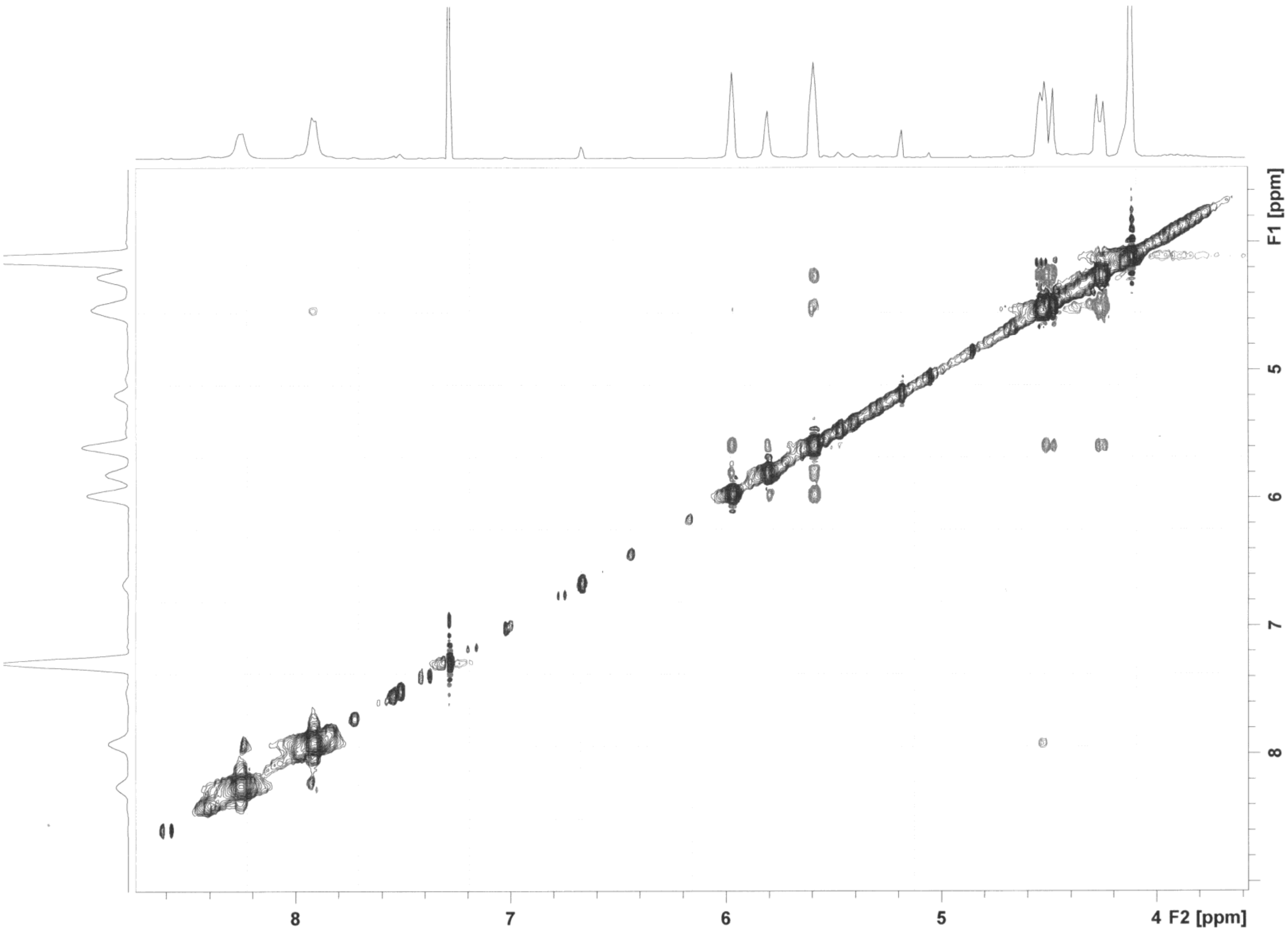

NOESY ($CDCl_3$) of pyridazine 5d.

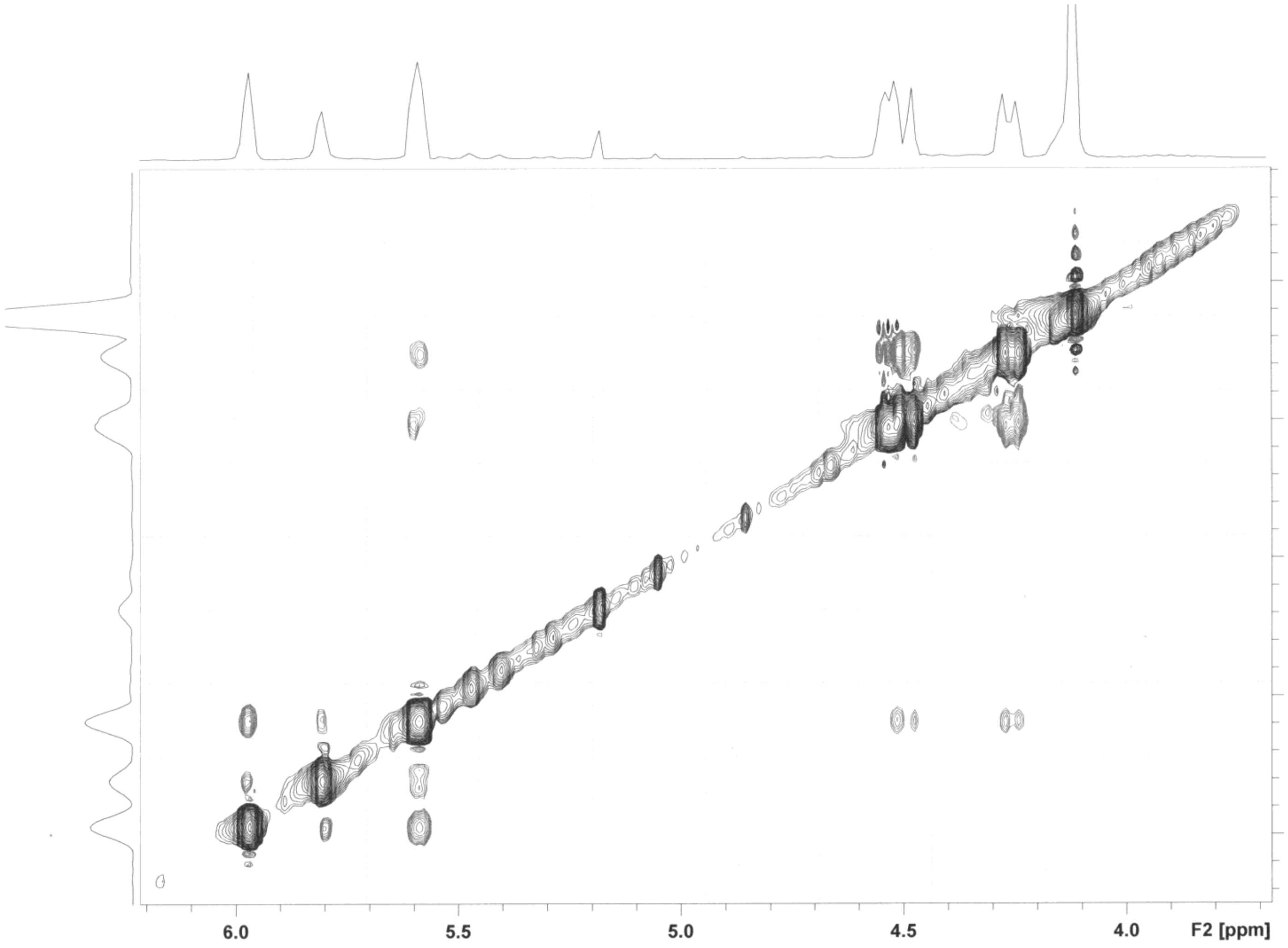

Expanded NOESY ($CDCl_3$) of pyridazine 5d.

X-Ray crystallography

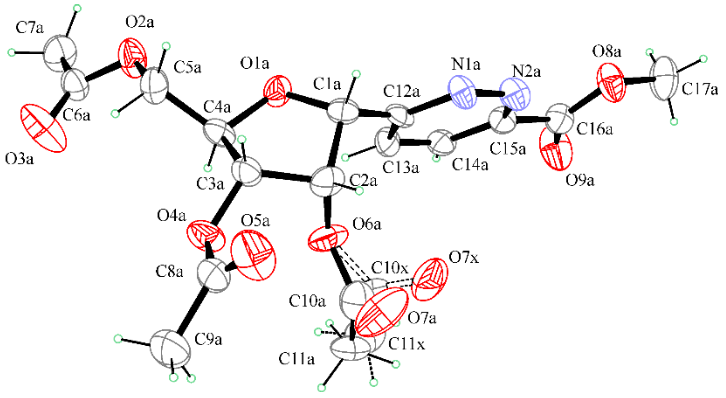

A

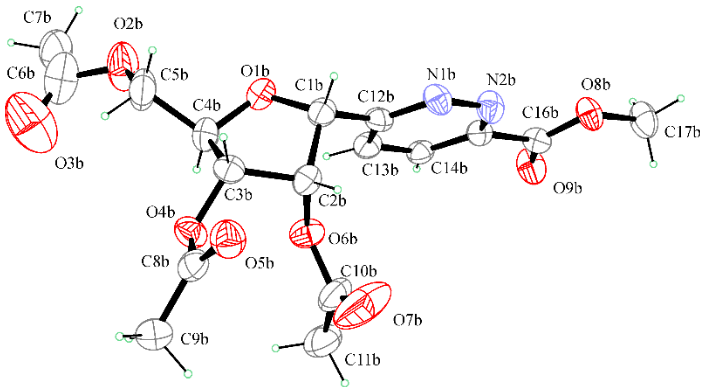

B

Figure S31. Ortep view of **5d**. The two independent molecules **A** and **B** are drawn a similar perspectic view. Thermal ellipsoids are drawn at 30% probability level. The minor part of disordered acetyl group in molecule **A** is drawn as open lines. As shown, in both molecules the eterocyclic ring is in the twisted conformation and the substituents at C1 and C4 display the mutually trans configuration typical of α-anomers.

Table S1. Crystal data and structure refinement details for **5d**.

	**5d**
Empirical formula	$C_{17}\,H_{20}\,N_2O_9$
Formula weight	396.35
T (K)	298(2)
λ (Å)	0.71073

Crystal system	orthorhombic
Space group	$P\,2_1\,2_1\,2_1$
a (Å)	5.783(2)
b (Å)	20.372(6)
c (Å)	33.116(8)
α (°)	90
β (°)	90
γ (°)	90
V (Å^3)	3901(2)
Z	8
D_{calc} (Mg/m^3)	1.350
μ (mm^{-1})	0.111
F(000)	1664
θ Range (°)	3.06 – 25.01
Reflections collected / unique [R(int)]	18825 / 6519 [R(int) = 0.1040]
Data / restraints / parameters	6519 / 50 / 541
Goodness-of-fit on F^2	1.014
Final R indices [I>2σ(I)]	R1 = 0.0847<br>wR2 = 0.1705
R indices (all data)	R1 = 0.2142<br>wR2 = 0.2228
Largest diff. peak and hole eA^{-3}	0.472 and -0.261
